# Engineered peroxisomes and patient-derived iPSC models as potential biomaterial platforms for precision neuroprotection in oxidative stress-driven central nervous system disorders

**DOI:** 10.1016/j.mtbio.2026.103752

**Published:** 2026-10-13

**Authors:** Csaba Matta, Yoon-Hee Cheon, Judit Vágó, Zoltán Mészár, Ee-Hyun Kim, Songling Jiang, Andrea Balogh, Melinda Zana, András Dinnyés, Eun-Jung Jin

**Affiliations:** a Department of Anatomy, Histology and Embryology, Faculty of Medicine, University of Debrecen, Debrecen, Hungary; b Department of Biomedical Materials Science, Graduate School of JABA, Wonkwang University, Iksan, Republic of Korea; c Integrated Omics Institute, Wonkwang University, Iksan, Republic of Korea; d Smart Convergence Materials Analysis Center, Wonkwang University, Iksan, Republic of Korea; e BioTalentum Ltd., Godollo, Hungary; f Department of Physiology and Animal Health, Institute of Physiology and Animal Nutrition, Hungarian University of Agriculture and Life Sciences, Godollo, Hungary; g Organelle Therapeutics Inc., Jeonbuk, Republic of Korea

**Keywords:** Peroxisome engineering, Artificial organelles, Patient-derived iPSCs, Biomaterials, Oxidative stress, CNS delivery, Kleefstra syndrome

## Abstract

Peroxisomes couple hydrogen peroxide metabolism to lipid homeostasis, making their functions relevant to neurological disease and to the design of intracellular catalytic biomaterials. However, the strength of disease-specific evidence and the functions reproduced by current materials vary substantially. This narrative review distinguishes modified biological peroxisomes from artificial and biomimetic platforms and evaluates how patient-derived induced pluripotent stem cell (iPSC) models could support their development. We examine membrane chemistry, substrate permeability, enzyme retention, catalytic activity, cell targeting, blood-brain barrier transport, three-dimensional tissue penetration, and intracellular fate. We separate the established causal role of peroxisomal defects in primary peroxisomal disorders from associative or unresolved findings in Alzheimer's disease, Parkinson's disease, and amyotrophic lateral sclerosis. Kleefstra syndrome is discussed as a hypothesis-generating neurodevelopmental model, without assuming a primary peroxisomal defect. Published artificial-organelle studies establish selected catalytic and delivery capabilities, but do not validate the integrated patient-specific CNS therapeutic strategy proposed here. We therefore outline a staged evaluation framework that combines materials characterization with mechanistic controls, differentiated neural cultures, multicellular models, animal pharmacology, safety, and manufacturing assessment. Comparison with conventional antioxidant carriers, plasmalogen supplementation, and PPAR agonists should determine whether compartmentalized catalysis offers a measurable advantage. We provide a platform classification, a disease-resolved evidence hierarchy, and a set of controls and measurable endpoints intended to make peroxisome-inspired constructs comparable across studies. Clinical development will require evidence of disease-relevant benefit beyond ROS reduction and an acceptable therapeutic window.

## Highlights

• Peroxisome engineering requires controlled transport and retained catalytic activity.
• Patient-derived neural models can test material function and disease relevance.
• Peroxisomal causality differs across neurological disease contexts.
• CNS entry, tissue penetration and intracellular activity need separate validation.
• Translation requires efficacy, safety and reproducible manufacturing evidence.

## Graphical abstract

A simplified concept linking a measured neural disease phenotype, a material with defined catalytic activity, and functional testing in patient-derived neural models. BBB delivery and safety remain independent requirements. The concept is proposed rather than clinically validated.

**Figure:**
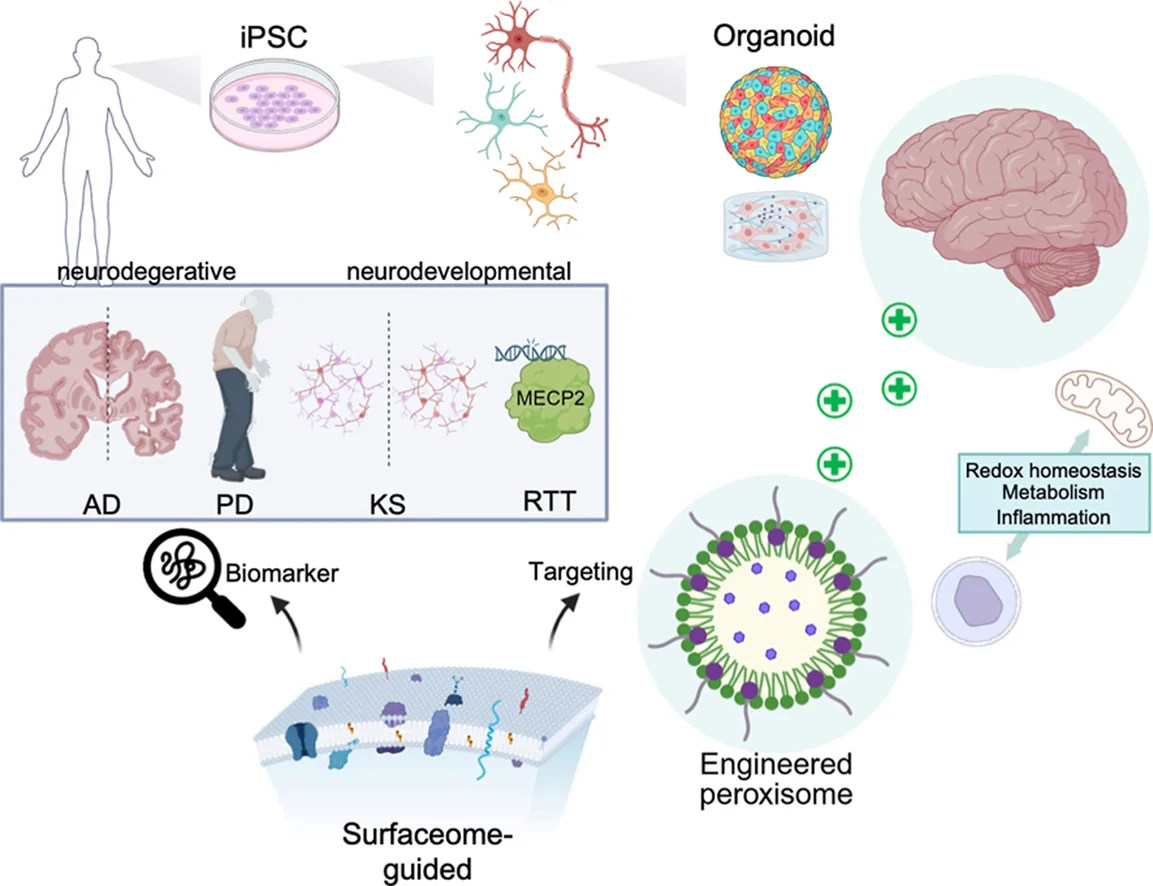

## 1 Introduction

Neurodegenerative disorders (NDD) such as Alzheimer's disease (AD), Parkinson's disease (PD), and amyotrophic lateral sclerosis (ALS) rank among the leading causes of disability and death in ageing populations, and their incidence is projected to rise steeply over the coming decades [1]. These conditions, together with related pathologies including frontotemporal dementia, Huntington's disease, and various leukodystrophies, affect distinct but partially overlapping neural networks. AD primarily targets brain regions involved in memory and cognition, leading to progressive decline in thinking, behaviour, and independence, and is the leading cause of dementia worldwide [2]. PD is characterized mainly by motor symptoms such as tremor, slowness, and rigidity due to degeneration of dopamine-producing neurons, although many patients also develop non-motor symptoms, including cognitive impairment and sleep disturbances [3]. ALS predominantly affects motor neurons in the brain and spinal cord, causing progressive muscle weakness, paralysis, and ultimately respiratory failure, with most patients dying within a few years of diagnosis [4]. In parallel, several neurodevelopmental syndromes like Kleefstra syndrome (KS) and Rett syndrome (RTT) with prominent cognitive and psychiatric features exhibit early-life circuit dysfunction that often evolves on a background of chronic cellular stress [5], [6].

Neurodegenerative and neurodevelopmental disorders differ in disease timing and the processes that a treatment would need to modify. Late-onset neurodegeneration involves progressive dysfunction or loss of established neural circuits [7], whereas KS primarily reflects disturbed developmental and epigenetic programmes [5], [8]. Patient-derived iPSC models are therefore especially useful for studying early developmental phenotypes. Modelling late-onset disease is more difficult because reprogramming resets several ageing-associated features. Maturation protocols, chronic challenges, or defined ageing manipulations can reveal selected phenotypes, but none should be assumed to reproduce normal human brain ageing [9].

Oxidative injury, mitochondrial dysfunction, protein homeostasis, and neuroinflammation interact in many neurological disorders, although their relative contributions differ [10]. These overlaps motivate investigation of shared cellular mechanisms without implying a common initiating lesion or a uniform therapeutic response. The developmental timing of an intervention also matters: a treatment that improves stress tolerance in mature neurons may not correct altered circuit formation [11].

Oxidative stress reflects disruption of redox control and/or oxidative damage when oxidant production exceeds the capacity of relevant protective systems [12]. Oxidized lipids, proteins, and nucleic acids have been reported in neurodegenerative disease, but a bulk oxidative-stress measurement cannot identify the initiating organelle or establish a causal mechanism [13]. Mitochondria, peroxisomes, lysosomes, and the endoplasmic reticulum participate in interacting metabolic and redox networks, so their contributions need to be resolved spatially and functionally [14].

Peroxisomes support very-long-chain fatty acid (VLCFA) metabolism, the initial steps of ether-lipid synthesis, and localized hydrogen peroxide handling. Defects in peroxisome biogenesis or individual peroxisomal enzymes cause primary neurological disorders [15]. In common neurodegenerative conditions, reported peroxisomal abnormalities are more heterogeneous and their causal position is less certain [16]. KS provides a complementary model in which a possible peroxisomal contribution can be tested, rather than assumed from sensitivity to a redox-active toxicant [5].

Clinical results with antioxidant strategies have been inconsistent. Limited target engagement, exposure, disease timing, and the physiological roles of ROS can all complicate interpretation [17]. Compartmentalized catalytic systems offer a way to test whether spatially controlled intervention improves a defined redox defect. Their theoretical selectivity must nevertheless be demonstrated rather than inferred from an organelle-related label [18].

Peroxisome-inspired biomaterials include enzyme-containing vesicles and catalytic hybrid assemblies designed to reproduce selected peroxisomal functions [19]. Antioxidant activity is presently a more established design objective than reconstruction of peroxisomal lipid metabolism. Patient-derived neural cultures, including neurons and glia, provide complementary systems in which to test material uptake, biochemical activity, and disease-associated phenotypes [20].

This narrative review links peroxisome biology with materials design and proposes a staged evaluation framework using patient-derived iPSC models and surfaceome-informed targeting (Fig. 1). It is a critical synthesis of selected examples, rather than a systematic review or meta-analysis. Published biological findings, demonstrated material capabilities, and prospective therapeutic applications are distinguished throughout. The integrated CNS strategy is a proposal for experimental validation, not an established treatment platform.

**Fig. 1:**
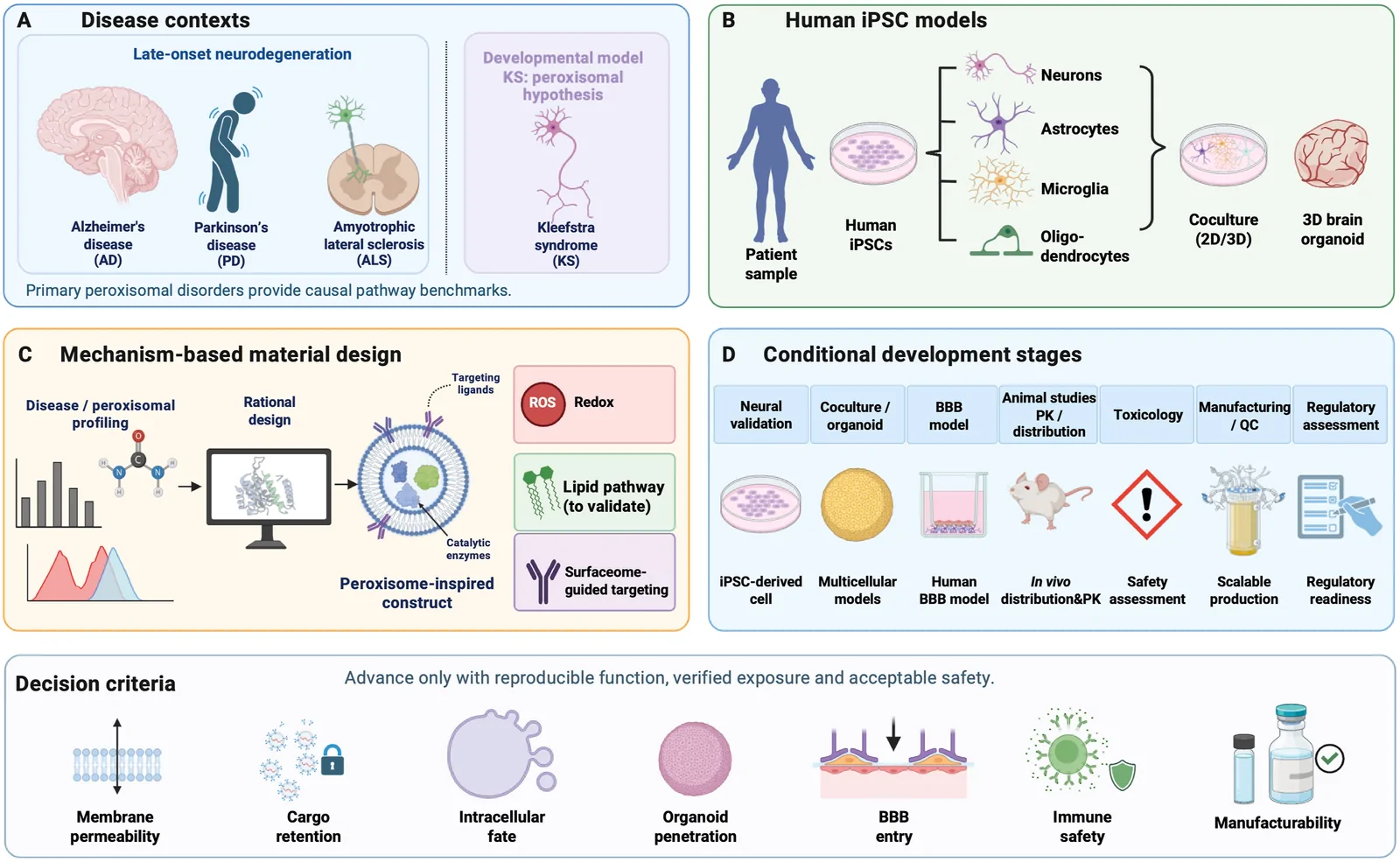
**Proposed evaluation framework for peroxisome-inspired CNS biomaterials.** (A) Disease selection distinguishes primary peroxisomal disorders, late-onset neurodegeneration, and KS as a hypothesis-generating developmental context. (B) Patient-derived neural systems enable disease phenotyping and measurement of peroxisomal function. Neural progenitor-derived spheroids and cerebral organoids are distinct model types. (C) Material composition, retained catalytic cargo, permeability, and validated surface ligands are selected for a defined mechanism. Antioxidant activity and lipid-metabolic replacement require different evidence. (D) Neural efficacy, BBB transport, animal pharmacology, safety, and manufacturing are evaluated through decision points with iteration between stages. Progression is conditional on reproducible target engagement, functional benefit, exposure, and acceptable safety. The diagram describes a proposed development process, not a validated therapeutic pipeline. Figure was created with BioRender.com.

## 2 Patient-derived iPSC models in neurodegeneration and rare neurodevelopmental disorders

iPSC technology enables patient-specific neural differentiation and controlled investigation of genetic disease mechanisms [8], [21], [22]. Regionally patterned organoids can capture selected features of cortical, midbrain, or other neural tissues, but the cell types and level of organization depend on the protocol [23]. Studies of AD and PD illustrate how disease-associated protein processing and cellular vulnerability can be investigated in these systems [24], [25], [26]. Such models reproduce aspects of pathology rather than the full clinical disorder.

Three-dimensional models also vary in how they are generated. The KS model reported by Balogh et al. consists of cortical neurospheres assembled from neural progenitor cells [5]; it should not be treated as interchangeable with a self-patterning cerebral organoid. Other neurodevelopmental models show altered neuronal network function or tissue organization [27], [28]. These distinctions matter when interpreting differences in penetration, cell composition, and treatment response.

Multicellular cultures and organoids allow measurement of neuron-glia interactions and network activity beyond single-cell survival [29], [30], [31], [32]. Their diffusion gradients, variable cell composition, and incomplete maturation can also confound apparent disease effects. Peroxisomal phenotypes therefore need direct measurement in each model rather than extrapolation from mitochondrial or inflammatory abnormalities reported in other neurological conditions [33], [34], [35].

As organoids undergo long-term maturation, they progressively develop diverse neuronal and glial subtypes, synaptic architecture, and metabolic gradients shaped by oxygen and nutrient diffusion, creating tissue-like microenvironments that are not captured in simple monolayer cultures [36]. These emerging niches support complex metabolic processes, including lipid oxidation and ROS handling that are central to peroxisomal function, and they enable interrogation of compartmentalized redox pathways and lipid metabolism in a human-relevant context, particularly in the setting of peroxisome dysfunction [34]. iPSC-derived neural cells from individuals with familial and sporadic AD, PD, ALS and related disorders reproduce disease-relevant phenotypes, including synaptic dysfunction, impaired axonal transport, mitochondrial defects, elevated ROS levels, and susceptibility to oxidative and excitotoxic insults [37], [38]. Beyond the relatively simple 2D monolayers and multicellular co-cultures, novel 3D brain spheroids and organoids successfully recapitulate higher-order properties such as neuron-glia cross-talk, neuroinflammatory signalling, and region-specific vulnerabilities that are difficult to access in primary human tissue or traditional animal models [29], [30], [39].

### 2.1 Strengths and limitations of iPSC-derived model systems

Because the term ‘iPSC-based model’ spans formats with markedly different biological properties, we distinguish these explicitly before considering their application to oxidative-stress modelling and engineered peroxisome testing (Table 1). Monoculture iPSC-derived neurons offer high reproducibility and scalability for mechanistic, cell-autonomous dissection of peroxisomal and redox phenotypes [40], since standardized differentiation and reagent protocols yield highly reproducible transcriptional signatures, but they retain an immature, foetal- or neonatal-like metabolic profile with limited oxidative phosphorylation and peroxisomal maturation. This “rejuvenation” of ageing signatures during reprogramming likely under-represents age-dependent phenotypes central to late-onset NDDs, and monocultures cannot capture non-neuronal contributions to redox and lipid homeostasis [41].

**Table 1:** Selection of iPSC-derived models for peroxisome-inspired material evaluation.

Model	Key strength	Main limitation	Most informative application	Representative readouts	Interpretation caveat
**Undifferentiated iPSC**	Patient genotype and renewable cellular starting material.	Lacks differentiated neural metabolism and function.	Cell-line quality control and initial compatibility testing.	Identity, genomic stability, viability, morphology and uptake.	Uptake or survival in iPSCs is not evidence of neural efficacy.
**iPSC-derived neuron**	Functional neuronal endpoints and subtype-specific vulnerability.	Cell-autonomous only; often immature or rejuvenated; culture-dependent stress responses.	Mechanistic testing of neuronal activity, exposure and protection.	Compartmental redox signals, oxidative damage, neurites, synapses and electrophysiology.	Late-onset disease features require explicit validation, not assumed ageing.
**iPSC-derived astrocyte**	Metabolic support, redox buffering and glutamate handling.	Maturation and reactivity depend on protocol and medium.	Evaluate metabolic support and effects on cocultured neurons.	Glutamate uptake, lipid profiles, cytokines, redox state and neuronal support.	Culture reactivity may not reproduce an *in vivo* disease state.
**iPSC-derived microglia**	Innate responses, phagocytosis and material recognition.	Strong dependence on environment, medium and maturation.	Compare surface coatings, uptake, activation and lysosomal handling.	Uptake, cytokines, phagocytosis, lysosomal function and persistence.	Systemic complement and adaptive immunity require additional assays.
**iPSC-derived oligodendrocyte**	Peroxisomal lipid metabolism and myelin-relevant biology.	Long maturation and protocol variability.	Test defined lipid defects and myelination-related outcomes.	Ether-lipid species, VLCFA processing, peroxisomal import and myelination.	Myelin protein expression alone does not establish functional myelination.
**Multicellular coculture**	Neuronal and glial interactions with controlled cell proportions.	Cell-specific mechanisms can be difficult to assign.	Determine whether benefit or toxicity depends on intercellular support.	Cell-resolved uptake, cytokines, redox metrics and network activity, spatially resolved redox/lipid readouts.	Average improvement can conceal opposing cell-type responses.
**Brain organoid or assembloid**	Spatial organization and multicellular network responses.	Variable composition, maturation, diffusion and vascular representation.	Test radial distribution and tissue-level response after verified exposure.	Carrier–cargo integrity by depth, active catalysis, cell-resolved injury and network measures.	Transport failure and catalytic failure are different explanations. Neurospheres are a distinct model type.
**BBB-on-chip or microfluidic BBB**	Controlled study of barrier interaction and transport.	Incomplete systemic physiology; endothelial identity can vary by differentiation method.	Distinguish endothelial binding from abluminal delivery of intact active material.	Barrier integrity, tracer permeability, mass balance and abluminal catalytic activity.	Endothelial uptake is not transcytosis. This is a complementary barrier model, not a neural efficacy model.

Note: Model selection is informed by Refs. [5], [21], [27], [29], [30], [39], [42], [43], [44], [45], [46], [47], [48], [49]. These references support model design and limitations, not validation of the integrated therapeutic platform. Use independent donors and differentiation batches, appropriate isogenic comparisons and analysis that accounts for donor/batch nesting. Technical wells or multiple organoids from one batch are not independent donors.

Abbreviations: iPSC, induced pluripotent stem cell; VLCFA, very-long-chain fatty acid; BBB, blood-brain barrier.

iPSC-derived astrocytes capture glial lipid handling, including peroxisome-dependent lipid mediator synthesis relevant to AD, PD, ALS, and RTT, although slower, batch-variable differentiation and heterogeneous maturation states (quiescent versus reactive) constrain cross-study comparability of peroxisomal readouts [21]. Although iPSC-derived microglia provide a useful platform for studying peroxisome-associated innate immune and neuroinflammatory signalling, they lack the tissue-resident environmental cues that shape microglial identity *in vivo*. Moreover, their basal activation state can vary substantially with culture conditions, potentially limiting reproducibility unless standardized co-culture systems or organoid-based models are used [20]. iPSC-derived oligodendrocytes are especially relevant given the central role of peroxisomes in myelin plasmalogen and VLCFA metabolism, making them essential for leukodystrophy-relevant, white-matter aspects of peroxisomal dysfunction [50]. These protocols are the most technically demanding and time-intensive, however, with pronounced donor-to-donor and batch variability that constrains screening throughput, as differentiation typically requires well over 100 days and yields remain inconsistent across cell lines [51].

2D cocultures partially restore cell-cell signalling and improve neuronal maturation relative to monocultures while retaining experimental tractability, but they lack the 3D architecture, gradients, and matrix cues that shape metabolic zonation and long-term maturation *in vivo* [52]. 3D cerebral and regionally patterned organoids most closely recapitulate human CNS cytoarchitecture and support emergent, network-level readouts alongside longer culture durations favouring maturation [23]. Their utility is nonetheless constrained by pronounced line-to-line and batch variability, non-uniform maturation, avascularity-driven hypoxic/necrotic cores that can confound oxidative-stress readouts, and typical absence of resident microglia unless specifically co-differentiated, alongside higher cost and lower throughput [53]. The extent to which organoids recapitulate the full complexity of adult, aged, or chronically stressed human CNS tissue also remains incompletely validated.

### 2.2 iPSC models of late-onset NDDs

Late-onset disease models need to distinguish genetic susceptibility from experimentally imposed injury. Progerin-induced ageing can reveal selected late-onset phenotypes, but it is a specific perturbation rather than a general substitute for ageing [9]. Similarly, gradual withdrawal of culture antioxidants can produce a tractable stress phenotype [42], but does not recreate the full aetiology of ALS or other neurodegenerative diseases. Baseline measurements, differentiation stage, culture medium, and exposure history should be reported so that therapeutic effects can be interpreted within the model's biological limits. Readouts should include functional outcomes alongside oxidative damage and survival.

### 2.3 iPSC models of Kleefstra syndrome and other neurodevelopmental disorders

KS is caused by EHMT1 haploinsufficiency, which perturbs chromatin regulation. Patient-derived neuronal studies link network abnormalities to enhanced NMDAR signalling [27], [54]. In cortical neurospheres, Balogh et al. reported altered cellular composition, reduced LDHA transcript abundance, and compound- and maturation-dependent toxicant sensitivity [5]. These observations justify testing metabolic vulnerability, but do not establish elevated basal ROS, abnormal peroxisomal lipid flux, or defective peroxisome biogenesis. A proposed connection between EHMT1 deficiency and peroxisomal pathways must therefore be tested using direct phenotyping and genetic rescue. Findings from other epigenetic disorders, including 7q11.23 syndromes, provide comparative developmental context rather than proof of a shared peroxisomal mechanism [55], [56].

Rett syndrome (RTT) provides another example of a neurodevelopmental disorder in which metabolic and redox mechanisms merit investigation. MECP2 mutations cause RTT [57], and studies have reported altered metabolite profiles and mitochondrial ROS production in relevant patient-derived systems [58], [59]. Lipid peroxidation and associated cell-death pathways have also been investigated [60]. These findings support disease-specific evaluation of redox mechanisms, but cannot establish peroxisomal causality in KS or other syndromes.

For neurodevelopmental models, the relevant question is whether an intervention improves a reproducible developmental or functional phenotype within an appropriate treatment window [61], [62]. Baseline abnormalities should be established before adding oxidative challenges. A greater response to treatment in a sensitized culture does not by itself establish disease selectivity or identify the organelle responsible.

KS thus serves as a hypothesis-generating example within a broader model hierarchy. Independent patient lines, appropriate controls, and mechanistic rescue are needed before a peroxisome-directed therapeutic rationale can be assigned to the syndrome.

## 3 Peroxisomes in central nervous system redox homeostasis and disease

Peroxisomes are single-membrane organelles that compartmentalize oxidative and lipid-metabolic reactions. They shorten VLCFAs and selected branched-chain substrates, initiate ether-lipid biosynthesis, and regulate hydrogen peroxide through oxidase activity and catalase-dependent decomposition [63]. Plasmalogen biosynthesis is completed through additional reactions in the endoplasmic reticulum. Peroxisomes therefore produce as well as remove oxidants, and their net effect depends on substrate supply, enzyme activity, and exchange with other cellular compartments [64].

In the CNS, peroxisomal functions contribute to myelin maintenance, membrane lipid composition, and neuronal and glial homeostasis [16]. Primary disorders differ in the affected biochemical pathway: defects of peroxisome biogenesis can impair several functions, whereas single-enzyme or transporter deficiencies produce more selective abnormalities. VLCFA accumulation and plasmalogen deficiency should therefore not be assumed to occur uniformly across all peroxisomal disorders. Their neurological manifestations establish the importance of specific peroxisomal functions, but do not show that antioxidant capacity alone is sufficient for rescue [65].

### 3.1 EHMT1, epigenetic dysregulation, and the rationale for peroxisomal involvement in Kleefstra syndrome

KS results from haploinsufficiency of EHMT1 (GLP), a histone H3 lysine 9 (H3K9) mono- and di-methyltransferase that represses transcription through deposition of repressive chromatin marks [66], [67]. Unlike primary peroxisomal biogenesis disorders, in which mutations directly impair peroxisomal protein import or enzymatic function, EHMT1 haploinsufficiency is expected to cause broad, cell-type- and context-dependent derepression of transcriptional programs rather than a discrete metabolic defect [54], [68], [69].

A potential connection between EHMT1 dysfunction and peroxisomal biology is therefore indirect and epigenetic. Loss of H3K9 methylation-dependent repression could alter expression of genes involved in lipid metabolism, peroxisomal and mitochondrial biogenesis, or redox regulation, particularly during neurogenesis and synaptogenesis. Consistent with a metabolically vulnerable phenotype, KS patient-derived iPSC neurons and organoids show altered energy metabolism, increased NMDA receptor-mediated network excitability, and enhanced susceptibility to oxidative and metabolic stress [5], [27]. However, these findings do not directly establish peroxisomal dysfunction: structural changes, enzyme activity, plasmalogen or VLCFA metabolism, and peroxisome–mitochondria contacts have not yet been systematically examined in KS models. Likewise, no study has directly tested whether peroxisome-targeted interventions can rescue KS-associated redox or metabolic phenotypes.

Accordingly, KS should not currently be regarded as a validated peroxisomal disease indication. Rather, it represents a plausible and experimentally tractable neurodevelopmental model in which epigenetically driven peroxisomal and lipid-metabolic dysregulation can be tested using iPSC-derived neuronal systems and multiscale functional readouts. Demonstrating that EHMT1 haploinsufficiency causally alters peroxisomal gene expression or function is an essential prerequisite for considering peroxisome-targeted therapies in KS.

In late-onset neurodegenerative disorders such as AD, PD, and ALS, peroxisomal abnormalities may instead represent dynamic, initially adaptive but ultimately maladaptive responses to chronic oxidative stress and proteinopathy. These changes involve peroxisome abundance, distribution, and organelle contact sites and are closely linked to astrocytic and microglial activation [10], [16], [70]. Thus, although peroxisomes may contribute to both developmental and degenerative brain disorders, their functions and therapeutic implications are context-dependent; strategies appropriate for KS should not be assumed to translate directly to AD or PD [7], [71].

### 3.2 Disease-specific evidence for peroxisomal involvement: causative, contributory, or secondary?

Distinguishing primary peroxisomal disease from potentially contributory peroxisomal abnormalities is essential for therapeutic interpretation. Whereas peroxisomal biogenesis disorders are directly caused by defects in peroxisomal import or enzyme function [68], [69], peroxisomal involvement in KS remains an experimental question. In common NDDs, including AD, PD, and ALS, temporal and perturbation-based evidence is required to distinguish causal contributions from adaptive responses or downstream injury; reduced ROS after treatment alone cannot resolve these alternatives.

In AD, post-mortem studies provide the strongest disease-specific evidence. Kou et al. reported altered VLCFA and plasmalogen profiles, increased somatic peroxisomal volume density, and loss of peroxisomes from tau-affected neuronal processes in vulnerable cortical regions [72]. These observations support peroxisomal involvement, but do not establish whether such abnormalities initiate pathology or emerge during progression alongside amyloid, tau, mitochondrial, and inflammatory changes. Peroxisome–mitochondria contact sites were not directly assessed. Thus, peroxisomal dysfunction is most cautiously viewed as a potential disease-modifying contributor in AD rather than an established primary driver.

Evidence in PD is less definitive and derives mainly from experimental models and limited human studies reporting altered lipid metabolism and possible disturbances in peroxisome–mitochondria interactions [16], [73]. Given the central roles of α-synuclein pathology and mitochondrial dysfunction, peroxisomal alterations in PD remain tentatively contributory or secondary. The evidence is weaker still in ALS, where proposed peroxisomal involvement is largely inferred from oxidative and lipid dysregulation in motor neurons and glia rather than from a defined, disease-specific peroxisomal lesion [74], [75]. Accordingly, peroxisome-directed approaches are best considered exploratory in ALS pending targeted mechanistic studies.

Peroxisomal metabolism also intersects with neuroinflammatory signalling in microglia and astrocytes, through effects on lipid mediators, ROS handling, glial polarization, and cytokine production [70], [71]. Glial peroxisome disruption in Drosophila causes axonal swelling and neuroinflammation, providing perturbation evidence for a role in CNS homeostasis, but not proof of an identical mechanism in human neurodegeneration [76]. These findings support cell-type-resolved studies that distinguish primary changes in peroxisomal metabolism from secondary inflammatory responses and clarify whether peroxisome-targeted interventions can modify disease-relevant phenotypes.

### 3.3 Mechanisms of peroxisome-mitochondria crosstalk in neuronal oxidative stress

Peroxisomes and mitochondria form an interconnected lipid–redox network that supports neuronal metabolic homeostasis and oxidative-stress resilience [14]. Their functions are coordinated through fatty-acid metabolism, hydrogen peroxide (H_2_O_2_) handling, membrane-lipid composition, shared fission machinery, and physical organelle contacts (Fig. 2). However, functional coupling, physical proximity, and directional molecular transfer should be distinguished experimentally rather than inferred from organelle co-localization alone.

**Fig. 2:**
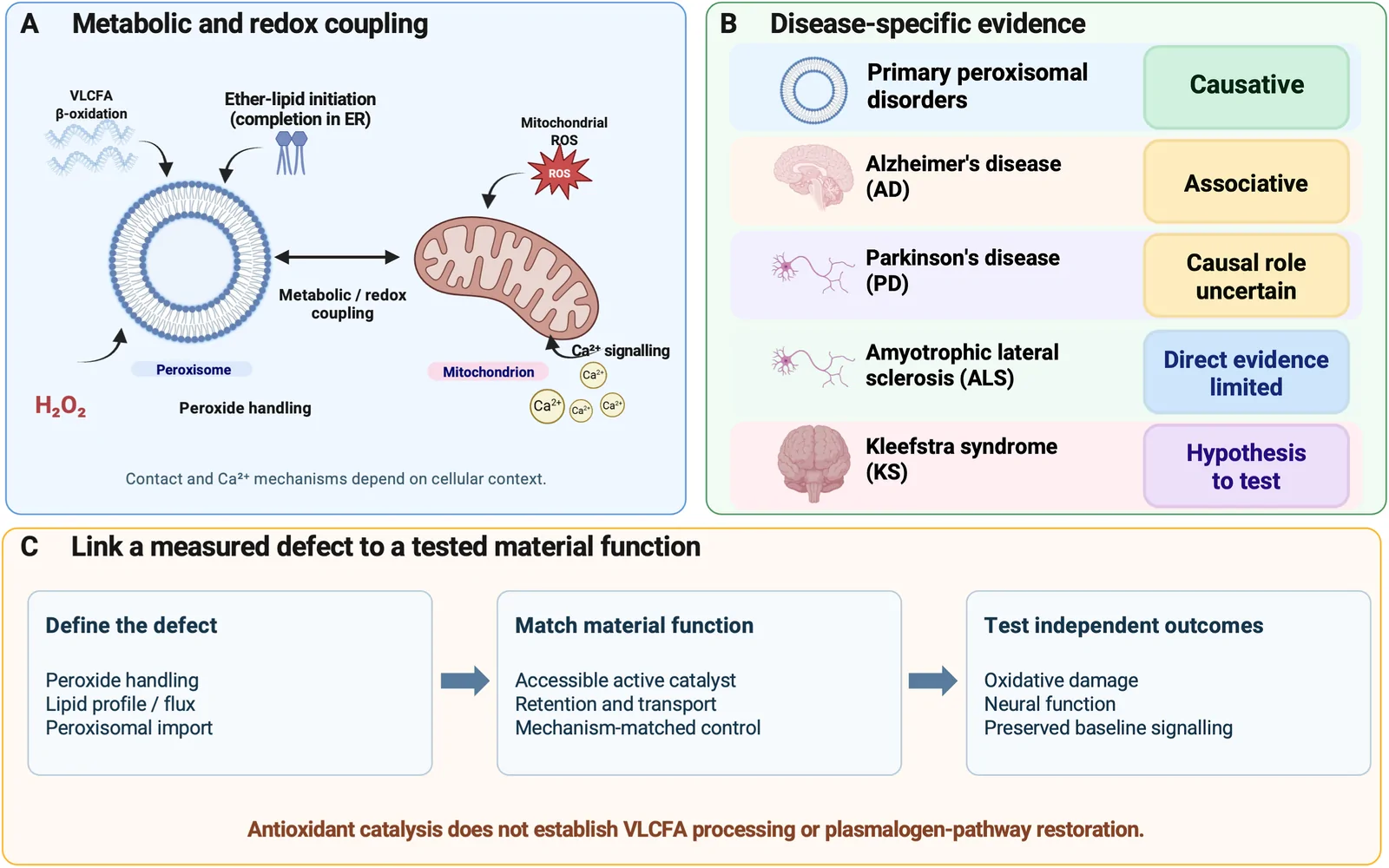
**Peroxisomal mechanisms and disease-specific interpretation.** (A) Peroxisomes contribute to fatty acid processing, the initial steps of ether-lipid synthesis, and H_2_O_2_ production and removal. Their interactions with mitochondria involve metabolic and redox coupling, while plasmalogen biosynthesis also requires the endoplasmic reticulum. Shared fission machinery and reported contacts do not establish universal directional substrate or Ca^2+^ transfer. (B) Peroxisomal defects are causal in primary peroxisomal disorders; AD evidence is largely associative, the causal position in PD and ALS remains uncertain, and KS requires direct peroxisomal phenotyping. (C) A disease-associated defect must be linked to a specific material function and an independent functional outcome. Demonstrated antioxidant catalysis should be distinguished from proposed lipid-metabolic restoration. Arrows indicate relationships or proposed evaluation steps, not established causal chains in all diseases. Figure was created with BioRender.com.

Peroxisomes initiate β-oxidation of very-long-chain and branched-chain fatty acids, generating shortened products that can enter mitochondrial metabolic pathways for further oxidation [63], [77]. Unlike mitochondrial β-oxidation, the first peroxisomal oxidation step transfers electrons to oxygen, producing H_2_O_2_ rather than directly generating ATP. Catalase normally limits this redox burden, but impaired peroxisomal oxidation or H_2_O_2_ detoxification may promote VLCFA accumulation and increase oxidative stress in adjacent mitochondria, compromising membrane integrity and electron-transport-chain function [34], [78].

Ether-lipid synthesis provides a second connection. Peroxisomal reactions initiate plasmalogen biosynthesis, which is completed in cooperation with the endoplasmic reticulum and influences neuronal, myelin, and mitochondrial membrane composition. Because plasmalogens can modulate membrane susceptibility to oxidation, impaired ether-lipid synthesis may increase mitochondrial oxidative vulnerability [75], [79]. Nevertheless, total plasmalogen abundance alone is insufficient to establish this mechanism; species-resolved lipidomics is required to define disease-relevant lipid alterations.

Crosstalk is also coordinated by physical contact sites and shared membrane-remodelling factors that may facilitate lipid exchange, metabolite transfer, fission, and organelle quality control, such as tethering complexes (e.g., ACBD2/ECI2, ACBD5–VAPA/B) and shared fission machinery (e.g., DRP1 with FIS1/MFF) [77], [80], [81]. Oxidative stress can generate a bidirectional amplification loop: defective peroxisomal ROS handling increases mitochondrial oxidative burden, whereas mitochondrial ROS can damage peroxisomal proteins and import machinery [82]. Mitochondrial Ca^2+^ handling is sensitive to these redox and metabolic changes and may influence permeability-transition and apoptotic thresholds; however, a universal direct peroxisome-to-mitochondrion Ca^2+^-transfer mechanism has not been established.

These mechanisms provide plausible routes through which peroxisomal dysfunction could amplify neuronal metabolic and oxidative vulnerability (Table 2). They do not, by themselves, establish disease-specific causality in AD, PD, ALS, or KS. A stronger therapeutic rationale requires evidence that a reproducible peroxisomal defect precedes or contributes to a disease phenotype and that selective correction of that defect improves relevant cellular or functional outcomes. General oxidative stress, altered organelle abundance, or rescue by a non-targeted antioxidant is insufficient to demonstrate peroxisomal causality.

**Table 2:** Disease-specific evidence for peroxisomal involvement in CNS disorders.

Disease or disorder	Direct evidence and source	What the evidence supports	What remains unresolved	Implication for material evaluation	Evidence category
**Alzheimer's disease**	Human postmortem lipid and organelle-distribution abnormalities, including VLCFA accumulation and plasmalogen reduction [72].	Association with neuropathological severity and altered peroxisomal distribution.	Temporal causality and reversibility. Contact-site disruption was not measured [72].	Select a reproducible biochemical phenotype and test whether correction improves neural function.	Human observational evidence; potentially contributory.
**Parkinson's disease**	Peroxisome-related lipid and quality-control mechanisms discussed in Refs. [16], [75].	A rationale to investigate peroxisomal participation in selected cellular states.	A primary peroxisomal lesion in typical PD is not established by these sources.	Measure peroxisomal function directly and distinguish it from mitochondrial-toxin injury.	Mechanistic literature; causal position uncertain.
**Amyotrophic lateral sclerosis**	Peroxisomal involvement is discussed within a broader oxidative and metabolic disease context [16].	A hypothesis requiring direct testing.	General ROS and lipid abnormalities do not establish a peroxisomal mechanism.	Demonstrate a peroxisomal phenotype and selective rescue before attributing treatment benefit.	Direct disease-specific evidence remains limited.
**Kleefstra syndrome**	NMDAR-associated network dysfunction [27]; altered LDHA expression and toxicant sensitivity in cortical neurospheres [5].	Developmental and metabolic vulnerability in the studied models.	Basal ROS elevation, peroxisomal lipid flux, and peroxisome biogenesis were not established by these observations.	Use direct peroxisomal assays, independent donors and isogenic comparisons before testing a causal rescue hypothesis.	Hypothesis-generating for peroxisomal involvement.
**Primary peroxisomal disorders**	Genetic and biochemical defects in biogenesis, transport or individual enzymes [45], [65], [69], [83].	Causality for the affected peroxisomal pathway.	Biochemical defects and reversibility differ by disorder and developmental stage.	Match the construct to the missing function. Antioxidant activity alone does not restore all lipid pathways.	Genetic and biochemical causal evidence.

Note: Evidence categories are descriptive and are not a formal GRADE assessment. Associations and general oxidative stress do not establish peroxisomal causality.

Abbreviations: VLCFA, very-long-chain fatty acid; PD, Parkinson's disease; ROS, reactive oxygen species; NMDAR, N-methyl-D-aspartate receptor; LDHA, lactate dehydrogenase A.

## 4 Peroxisome engineering and artificial organelles

### 4.1 Classification and conceptual framework of peroxisome-engineering platforms

Peroxisome engineering is used here as an umbrella for modifying biological peroxisomes or constructing materials that reproduce selected peroxisomal functions. A natural peroxisome is an intact endogenous or isolated biological organelle. An engineered biological peroxisome retains biological organelle architecture but has intentionally altered cargo, membrane components, localization, or function. An artificial peroxisome is a synthetic or hybrid catalytic system designed to emulate a defined peroxisomal reaction; published examples include membrane-bounded nanoreactors and non-vesicular catalytic assemblies. Biomimetic describes the particular biological feature reproduced, such as a membrane interface or H_2_O_2_-decomposing activity, and is not a separate mutually exclusive material class. Nanoorganelle denotes a nanoscale artificial functional compartment and does not establish peroxisomal identity. Conventional antioxidant nanocarriers may share enzymes, catalysts, and targeting chemistry with these systems. The distinction depends on demonstrated compartmentalized function, transport, and retention rather than nomenclature alone [19], [84], [85], [86], [87]. Table 3 compares these platform classes without implying functional equivalence or superior clinical maturity.

**Table 3:** State-of-the-art in peroxisome engineering.

Study and evidence role	Matrix material	Enzymatic cargo or catalyst	Targeting strategy	Transport or assembly feature	Model system tested	Reported efficacy in neural models	Interpretation and verification need
**Artificial-peroxisome benchmark**[85]	PMOXA–PDMS–PMOXA triblock-copolymer polymersomes incorporating OmpF channel proteins	Encapsulated Cu/Zn-superoxide dismutase (SOD) with lactoperoxidase (LPO); catalase (CAT)	Do not infer a targeting ligand from the term artificial peroxisome.	OmpF-mediated substrate transport; Substrate access with retention of macromolecular catalyst is the relevant design principle.	HeLa cells exposed to oxidative stress.	A patient-derived neural efficacy result is not established by the supplied summary.	Do not assign a neural efficacy value or specific ligand until the primary paper is checked.
**Targeted nanoorganelle benchmark**[19]	PDMS_29_-b-PMOXA_10_ diblocks, with PEG_4_–N_3_ end-functionalized chains for click coupling.	Encapsulated lactoperoxidase (LPO).	Arginine-rich cell-penetrating peptide (RR)_4_(KLL)_2_KGKC; not a CNS-selective receptor ligand.	Melittin pores. Optimized CPP-bearing vesicles had a reported hydrodynamic diameter of 140 ± 1 nm.	K562 myelogenous leukaemia cells challenged with H_2_O_2_.	No neural model tested. ESR indicated intracellular ROS attenuation in K562 cells.	Direct material/cargo/transport benchmark; cell penetration does not establish BBB transport or neural selectivity.
**Non-vesicular artificial peroxisome**[87]	Branched DNA with pH-responsive i-motif sequences and ceria nanoparticles.	Ceria with SOD-, catalase- and peroxidase-like activities; not encapsulated natural enzymes.	Intracellular assembly; no CNS-cell-selective ligand established here.	Acidic endolysosomal conditions trigger larger assemblies and support retention.	Living-cell oxidative-stress experiments.	The study establishes cellular ROS protection, not patient-derived AD/KS neural efficacy.	An experimental example of programmable assembly and selected redox catalysis, not full peroxisome replacement.
**Non-CNS therapeutic example**[88]	Mesenchymal stem cell-derived extracellular vesicles loaded with Mn_3_O_4_ nanozyme and further cloaked with platelet membrane (Mn@LPEV)	Mn_3_O_4_ nanozyme with immobilized lactate oxidase (LOX)	Platelet-membrane-based biomimetic targeting combined with a lactate-responsive delivery design for injured myocardium	MSC-EV encapsulation of Mn_3_O_4_ followed by platelet-membrane cloaking and LOX immobilization	Myocardial injury and heart-repair context.	Cardiac benefit cannot be treated as a neural or BBB efficacy result.	Relevant *in vivo* indication context, but detailed material claims must be checked against the primary paper.
**Peroxisomal localization precedent**[89]	Supramolecular structures formed from pyrene-labelled peptide amphiphiles.	Fluorescent nanoprobe; not a therapeutic multienzyme compartment.	C-terminal Ser–Lys–Leu (SKL) peroxisome-targeting sequence; sequence and assembly structure affect localization.	Self-assembly and localization.	Mammalian-cell localization; the accessible primary abstract does not specify the cell line.	Localization is not evidence of neuroprotection or metabolic rescue.	A targeting-method precedent, not an artificial-peroxisome efficacy benchmark.
**Mitochondrial design precedent**[90]	Designed multiblock peptide amphiphiles forming ellipsoidal nanoparticles.	Bioactive peptide architecture; no peroxisomal enzyme cargo.	Mitochondrial VDAC1–HK-II complex.	Self-assembly, cellular uptake and organelle-associated interaction targeting.	Melanoma inhibition.	Not a neural efficacy study and not direct peroxisome evidence.	Antitumour disruption of a protein interaction is not equivalent to restoring neuronal homeostasis.
**Review-level context**[86]	Multiple membrane-cloaked catalyst classes are reviewed.	Heterogeneous natural enzymes and enzyme-mimetic catalysts.	Several membrane sources and targeting approaches. (e.g., neutrophil- or erythrocyte-membrane-based delivery)	Review synthesis; no single platform or uniform transport measurement.	Multiple biomedical applications reviewed. (e.g., 3xTg-AD mice, MPTP-induced PD mice)	Neural studies discussed in the review must be traced to their individual primary papers.	This article is a review, not one experimental *in vivo* benchmark.

Note: This is a structured comparison of selected examples, not a systematic ranking. Reviews and non-peroxisomal precedents are identified explicitly.

Abbreviations: PMOXA, polymethyloxazoline; PDMS, polydimethylsiloxane; PEG, polyethylene glycol; CNS, central nervous system; CPP, cell-penetrating peptide; ESR, electron spin resonance; ROS, reactive oxygen species; AD, Alzheimer's disease; KS, Kleefstra syndrome; MSC-EV, mesenchymal stem cell-derived extracellular vesicles; BBB, blood-brain-barrier; MPTP, 1-methyl-4-phenyl-1,2,3,6-tetrahydropyridine.

Genetically encoded tools can perturb biological organelles to test their function. Watanabe et al. developed engineered phospholipase A/acyltransferase systems for organelle defunctionalization [91]. These tools manipulate organelle integrity rather than replace peroxisomal function, and do not establish therapeutic efficacy in patient-derived neural models. Complementary approaches that modify endogenous peroxisomal enzymes should likewise be distinguished from delivery of an isolated organelle or a synthetic nanoreactor.

Artificial organelle studies demonstrate selected forms of intracellular catalysis and material-controlled transport, but not full peroxisomal function. Maffeis et al. used PDMS-PMOXA diblock polymersomes containing lactoperoxidase, melittin pores, and an arginine-rich cell-penetrating peptide, which improved intracellular ROS handling in K562 leukaemia cells; this provides a permeability-and-uptake benchmark rather than a neural or BBB efficacy result. Current proof-of-concept evidence otherwise derives from simplified mammalian cell systems exposed to oxidative stress and, more recently, an *in vivo* cardiovascular ischaemia–reperfusion model [19], [85], [88]. To date, engineered or artificial peroxisomes have not been evaluated in patient-derived iPSC models of AD, PD, ALS, KS, or other CNS disorders discussed here, and none of these studies reproduces the genetic background, neural maturation state, glial interactions, extracellular matrix, or chronic exposure conditions of human iPSC-derived neurons and brain organoids. No benchmark study therefore validates the full proposed combination of a peroxisome-inspired construct, patient-derived neural tissue, selective CNS delivery, and disease-relevant functional rescue. The gap is an absence of demonstrated integration rather than a documented failure of clinical translation, and this review accordingly presents a conceptual and experimental framework at the intersection of organelle engineering and iPSC-based neural disease modelling rather than evidence of completed therapeutic benchmarking.

### 4.2 Material chemistry, selective permeability, and catalytic retention

The membrane of an artificial peroxisome is a reaction interface, not simply an encapsulation shell. Liposomes offer lipid composition and fluidity that can be adjusted through phospholipid and cholesterol selection, but leakage and oxidation must be evaluated for each formulation. Amphiphilic block copolymers permit control of membrane thickness, hydration, packing, and degradation. Relevant materials discussed in the nanoreactor literature include PEG-b-PLA and PEG-b-PCL polyester systems, PMOXA-b-PDMS-b-PMOXA triblocks, and PMPC-b-PDPA [92], [93], [94]. PMPC provides a zwitterionic hydrophilic block, whereas protonation of the PDPA block can alter membrane organization under acidic conditions. These are material design options, not evidence that every polymer has been validated as a CNS peroxisome substitute.

Catalytic performance depends on the balance between substrate transport and intraluminal reaction. Membrane permeability, surface area, substrate partitioning, and the concentration gradient determine substrate entry, while accessible active enzyme determines consumption. If transport is slower than catalysis, adding enzyme may increase loading without improving overall reaction rate. Apparent kinetic parameters measured for a nanoreactor therefore combine membrane transport and enzyme activity and should not be interpreted as intrinsic enzyme constants. Permeability should be assessed under the relevant pH, ionic strength, temperature, and protein conditions. Paired measurements with free enzyme, intact compartments, and deliberately permeabilized compartments can help distinguish diffusion limitation from enzyme inactivation [93], [94].

Small-molecule transport can be supported by permeable membrane chemistry, engineered defects, or inserted pores such as OmpF [92], [93], [94]. Pore incorporation requires attention to insertion efficiency, orientation, membrane compatibility, and leakage. H_2_O_2_ access cannot be generalized to all ROS: superoxide is charged and has different transport constraints, while hydroxyl radicals react too locally and rapidly to be treated as freely diffusing cargo. Encapsulated superoxide dismutase (SOD) is useful only if superoxide reaches it, and the H_2_O_2_ it generates must be removed by sufficient catalase or another compatible system. Fatty acyl-CoA substrates also cannot be assumed to traverse a porin as freely as small soluble metabolites. Studies should report active enzyme loading, removal of unencapsulated enzyme, substrate-dependent reaction rates, enzyme leakage, membrane integrity, and catalytic persistence. Lipid-metabolic replacement additionally requires verified substrate access, cofactors, sequential reactions, and product export [63].

### 4.3 Programmable and stimuli-responsive organelle biomaterials

Programmable supramolecular materials broaden the organelle-engineering toolkit. Zhang et al. reported multiblock peptide amphiphiles that assemble into ellipsoidal nanoparticles and target the mitochondrial VDAC1-HK-II interaction in a melanoma setting [90]. This is a precedent for modular assembly and organelle-associated molecular recognition, not direct evidence of peroxisomal targeting or neuronal protection. Disrupting a mitochondrial interaction to kill tumour cells is also mechanistically distinct from restoring neural homeostasis. For peroxisome-inspired constructs, a proposed responsive design should specify the trigger, its operating range, the material change, and the resulting effect on catalytic activity. Changes in pH, oxidant concentration, or enzyme exposure may alter permeability, assembly, or ligand accessibility, but a trigger-response relationship must be demonstrated for the formulation rather than inferred from the term biomimetic (Fig. 3). Table 3 distinguishes experimental examples, transferable design precedents, and review-level context.

**Fig. 3:**
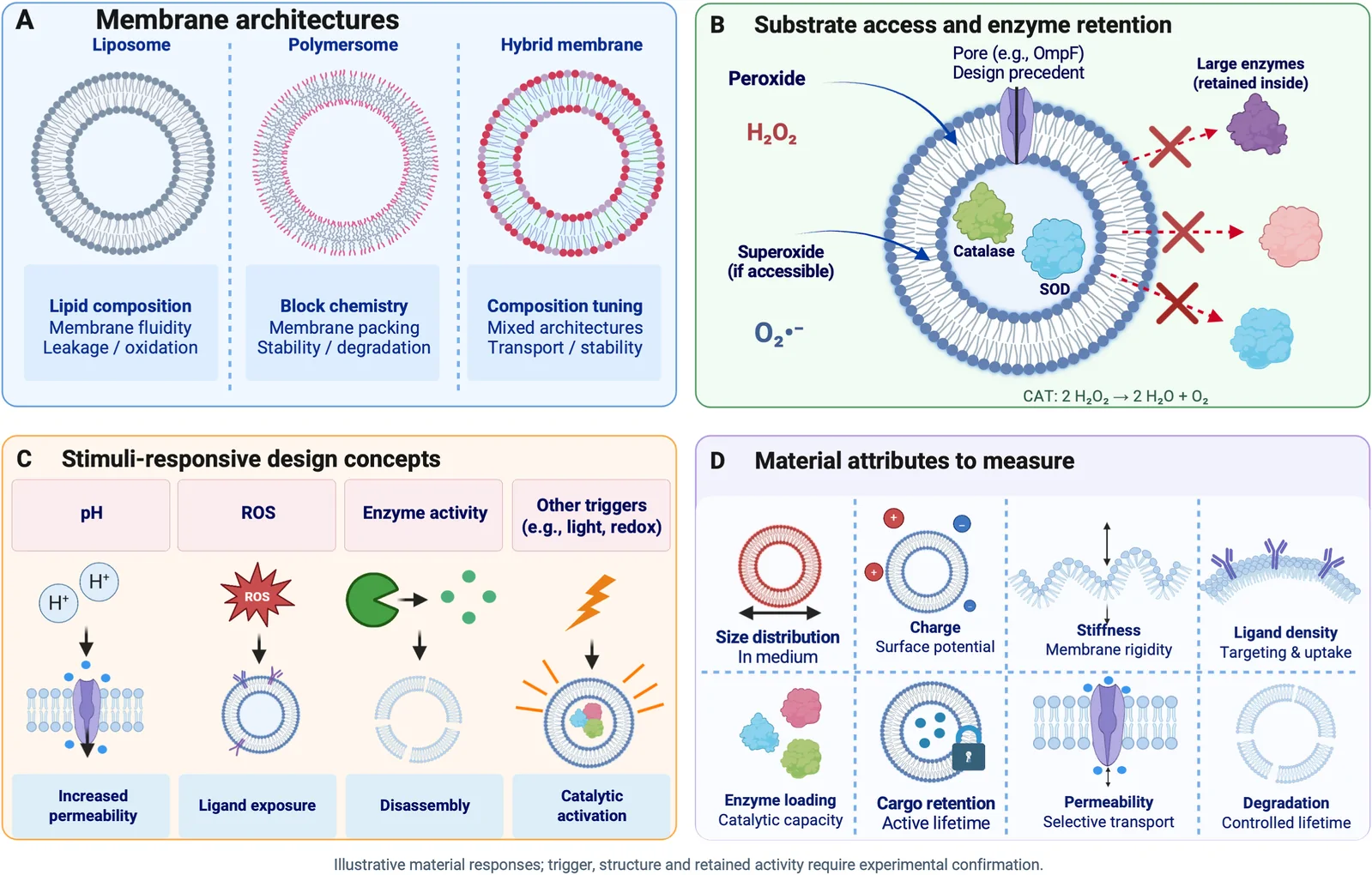
**Material design and catalytic transport in artificial peroxisomes.** (A) Liposomal, polymeric, and hybrid membranes provide different options for composition, permeability, stability, and degradation. These properties require measurement for each formulation. (B) H_2_O_2_ must access catalase while active enzyme is retained. OmpF illustrates a pore-forming design precedent, not a validated component of every platform. SOD converts accessible superoxide to H_2_O_2_, requiring sufficient downstream peroxide removal. H_2_O_2_, superoxide, and hydroxyl radical are distinct chemical species with different transport and reaction behaviour. (C) Stimuli-responsive materials require an experimentally demonstrated link between trigger, structural response, and catalytic performance. (D) Relevant measurements include hydrodynamic size, charge, membrane mechanics, ligand density, active loading, leakage, permeability, and degradation. No universal size or charge optimum for CNS delivery is implied. Figure was created with BioRender.com.

Engineered and artificial peroxisomes may offer conceptual advantages over conventional antioxidants or pathway-restricted small molecules by compartmentalizing enzymes and supporting localized redox or lipid-metabolic reactions. Their modular design permits adjustment of size, membrane permeability, enzyme loading, degradation, and targeting ligands, including peptides, antibodies, or cell-membrane cloaks to potentially direct activity towards vulnerable neuronal populations or reactive glia [19], [95], [96]. However, neither multienzyme loading nor surface functionalization alone establishes physiological peroxisomal activity or cell-type selectivity. For example, catalase/SOD-containing systems do not reproduce VLCFA β-oxidation or plasmalogen synthesis unless the requisite metabolic machinery and flux are demonstrated, and a targeting ligand does not itself establish selective uptake by diseased CNS cells.

Accordingly, any claimed advantage over a conventional antioxidant carrier should be supported by matched comparisons of catalytic dose, cellular exposure, intracellular persistence, functional outcome, and toxicity. Translational development will require evidence that candidate materials can be reproducibly manufactured, reach relevant cells at an active dose, retain their intended function, and provide benefit within a safe exposure range [97], [98]. Human iPSC-derived neural models can test key aspects of target-cell engagement and disease-relevant efficacy, but cannot replace systemic pharmacology, biodistribution, and safety studies [99].

Thus, these platforms should be viewed as potentially adaptable organelle-directed biologic therapeutics for late-onset NDDs and KS, subject to rigorous demonstration of delivery, mechanism-specific activity, and therapeutic benefit [88], [100]. Challenges include scalable and reproducible manufacturing of complex nanoassemblies, long-term safety, immune recognition, and reliable and efficient targeting to the human brain, particularly crossing or bypassing the blood–brain barrier (BBB) without off-target accumulation in peripheral organs and, critically, a substantially more detailed safety profile than has so far been articulated for this emerging therapeutic class, which we address next.

### 4.4 Safety considerations for engineered peroxisomes

Safety assessment of engineered peroxisomes should address construct immunogenicity, complement activation, redox perturbation, interference with endogenous organelle function, and the fate of cargo and carrier materials [84], [95], [97]. Protein cargo, biological targeting ligands, and non-native polymeric or membrane components may provoke immune recognition. Surface properties such as charge, PEGylation, and residual manufacturing impurities can also influence complement activation, opsonization, and repeat-dose clearance. Autologous or syngeneic membrane cloaking may reduce, but does not eliminate, these risks [96], [101].

A central mechanism-specific concern is that ROS are also physiological signalling molecules. Low concentrations of H_2_O_2_ regulate redox-sensitive proteins involved in neuronal differentiation, synaptic plasticity, and adaptive stress responses; peroxisome-like artificial organelles can measurably alter NRF2-associated antioxidant signalling after oxidative challenge [17], [19], [102], [103]. Excessive or poorly localized ROS scavenging could therefore impair neuronal signalling, suppress protective Nrf2-dependent or mitohormetic responses, and alter glial functions that require controlled ROS production, including microglial activation and host defence [70], [104], [105]. Candidate constructs should consequently be designed for tuneable, threshold-limited, or spatially restricted activity rather than maximal ROS removal, and evaluated using both oxidative-damage and redox-signalling endpoints. Peroxisomes themselves are redox-signalling organelles, with H_2_O_2_ acting as a diffusible second messenger; this further argues against treating ROS reduction as an unqualified therapeutic endpoint.

Peroxisome-mimetic activity may also perturb endogenous peroxisomal and mitochondrial homeostasis. Altered local substrate availability, cofactor balance, or redox flux could affect peroxisome–mitochondria crosstalk and adaptive regulation of peroxisome abundance. Constructs that introduce lipid-metabolizing functions require scrutiny because native peroxisomes are essential for VLCFA metabolism and plasmalogen biosynthesis; unintended changes in lipid flux could affect membrane composition and myelin maintenance [63], [65], [106].

Finally, loss of compartmentalization, enzyme leakage, and intracellular persistence are construct-specific risks. Shell destabilization during extracellular or lysosomal processing could release active cargo outside the intended compartment, while polymer, lipid, or other degradation products may be cytotoxic or pro-inflammatory depending on their dose and clearance [84], [95], [107]. Slowly degrading materials may accumulate after repeated dosing and disrupt lysosomal or autophagic function in neurons and glia, although these long-term effects remain uncharacterized for engineered peroxisomes [86], [108], [109].

Preclinical assessment should therefore combine standardized iPSC-derived neural models with repeat-exposure studies measuring uptake, intracellular distribution, persistence, degradation, redox signalling, lipid homeostasis, and cell-type-specific functional outcomes [110]. These systems can identify neural-cell liabilities, but systemic pharmacokinetics, biodistribution, repeat-dose toxicology, and immunotoxicology must be established *in vivo* before clinical translation.

## 5 Peroxisome-inspired biomaterials for CNS applications

Peroxisome-inspired platforms extend intracellular catalytic biomaterials into a disease area in which lipid and redox functions are closely coupled [80], [87], [106], [111], [112]. The relevant design task is to match a measured cellular defect to a defined material function. For example, H_2_O_2_ decomposition can be tested directly with a catalase-based nanoreactor, whereas correction of a VLCFA or ether-lipid defect requires different biochemical components and flux measurements. These objectives should not be bundled into a generic claim of peroxisome restoration [63], [113].

The comparison with other CNS biomaterials is informative when it concerns a specific material-biology relationship. Hydrogels and scaffolds can alter matrix mechanics, local factor presentation, and inflammatory responses [111], [114]. Intracellular catalytic constructs instead require control of membrane transport, reaction rate, localization, and persistence. Both approaches can affect surrounding cells, so spatial confinement does not guarantee absence of off-target or network-level effects [82], [115].

Brain delivery requires several separate events: survival in circulation, interaction with the BBB, passage beyond the vascular endothelium, distribution through parenchyma, and uptake by the intended cell population. Particle size, shape, charge, deformability, colloidal stability, and the acquired protein corona can influence each event differently [116], [117], [118]. Evidence for a carrier or an unloaded vesicle cannot be transferred automatically to the corresponding multienzyme-loaded formulation.

### 5.1 Material-driven BBB transport and brain entry

BBB delivery should be optimized for each defined formulation rather than against a universal particle-size or charge threshold. Particle size, shape, surface charge, PEG density and chain length, stiffness, and deformability can each affect protein-corona formation, circulation, endothelial interactions, uptake, and intracellular trafficking [116], [118]. Near-neutral or mildly anionic surfaces may reduce nonspecific interactions, whereas strongly cationic surfaces can increase endothelial binding but also membrane injury, peripheral clearance, and endothelial retention. The effects of deformability and membrane cloaking are likewise context-dependent and should be measured directly for the intended carrier and transport route.

Receptor-mediated transcytosis provides a potential delivery route, but endothelial uptake does not itself establish brain entry. Constructs targeting the transferrin receptor, insulin receptor, LRP1, or related endothelial pathways should be compared across ligand affinity, valency, density, orientation, and receptor recycling behaviour. LRP1 is of particular interest here because it operates on both sides of the problem addressed in this review: it mediates bidirectional transcytosis at the brain endothelium, including amyloid-β efflux [119], and within the oligodendrocyte lineage it is required for peroxisome biogenesis, cholesterol homeostasis, and timely CNS myelination and white matter repair [120]. A single receptor may therefore, in principle, support both barrier crossing and preferential engagement of the myelin-producing compartment in which peroxisomal function is most clearly rate-limiting, although this convergence is inferred from separate literature and has not been exploited in one construct. Excessive binding avidity can favour endothelial retention or lysosomal sorting rather than abluminal release and parenchymal exposure; intermediate receptor engagement may be more compatible with productive transport [121]. A sequential design could combine a BBB-shuttle ligand with a second ligand for preferential uptake by the relevant neural or glial population, but both transport and cell specificity require empirical validation.

BBB studies should therefore measure barrier integrity, tracer permeability, particle mass balance, endothelial retention, and recovery of intact, catalytically active material from the abluminal compartment. Human iPSC-derived neurovascular models, BBB-on-chip systems, and integrated brain-organoid platforms can support this evaluation, but should be complemented by *in vivo* biodistribution and pharmacokinetic studies. Intranasal, local, or other barrier-bypass routes avoid different delivery constraints and require separate assessment of CNS distribution, exposure, and safety [98], [122].

### 5.2 Delivery, intracellular fate, and immune interactions in 3D neural models

Crossing the BBB addresses only the first delivery barrier. Within 3D neural tissues, engineered peroxisomes must diffuse through the extracellular matrix (ECM), reach relevant cells, retain catalytic integrity during uptake and intracellular trafficking, and attain a subcellular location compatible with their proposed mechanism. Based on principles established in other 3D matrix-containing culture systems, diffusion is expected to compete with matrix binding, cellular uptake, aggregation, and degradation; larger hydrodynamic size, strong ECM interactions, or high cellular sequestration may therefore promote peripheral retention and reduce exposure in the organoid core [123], [124]. Effective diffusion will depend on matrix composition, organoid radius, cell density, exposure duration, and the fraction of intact active material at each depth. Under a diffusion-limited approximation, the time required to reach a given distance increases with the square of that distance; uptake and matrix binding may further steepen radial concentration gradients. ECM heterogeneity provides an important biological context for these effects, but does not substitute for formulation-specific transport measurements [125]. More broadly, dense and heterogeneous ECM and nonspecific glial uptake can restrict nanoparticle access to deeper tissue and intended neuronal targets.

Radial fluorescence profiles should be corrected for optical attenuation and supported by section-based or cell-resolved measurements [126], [127]. Dual labelling of carrier and cargo can help distinguish intact constructs from cargo dissociation [128]. Bath exposure, perfusion, and microinjection address different experimental questions: microinjection can establish local activity after direct deposition but bypasses surface penetration and therefore cannot validate tissue diffusion or systemic delivery [126].

Cellular internalization and functional delivery should also be assessed separately. Uptake route is formulation- and cell-type-dependent, influenced by particle size, shape, stiffness, surface chemistry and charge, ligand identity and density, and the biological corona [129], [130]. Endocytosed material may remain functional within endosomal compartments if substrates are accessible, whereas cytosolic access is necessary only for mechanisms requiring it. Accordingly, endosomal escape is not a universal prerequisite for intracellular nanoreactors, but where cytosolic activity is intended, pH-responsive, fusogenic, or membrane-active designs must be evaluated for escape efficiency, carrier integrity, and membrane injury [131], [132]. Nanoparticles commonly enter endosomal compartments and can subsequently be trafficked to lysosomes or recycled; endosomal uptake alone should not be interpreted as successful subcellular delivery.

The required intracellular destination should follow the proposed reaction mechanism. Localization near native peroxisomes does not demonstrate membrane fusion, matrix import, or restoration of endogenous peroxisomal metabolism. In particular, PTS1- or PTS2-dependent import pathways direct soluble protein cargo through PEX receptors and cannot be assumed to import an intact nanoparticle [133]. Consistent with this, the SKL-tagged pyrene–peptide amphiphile nanoprobes described by Kim et al. achieved peroxisome-selective localization in mammalian cells, modulated by SKL terminal accessibility, peptide α-helicity, and fibrillar morphology, but demonstrate organelle-level targeting specificity rather than matrix import of the assembly [89] (Table 3). Peroxisome-mimetic systems may instead act as autonomous cytosolic nanoreactors, or use lipid anchors, membrane-inspired motifs, or interaction-targeting ligands to promote proximity to endogenous peroxisomes, mitochondria, or their contact sites [90], [134]. Claims of lipid-metabolic rescue should therefore be supported by functional metabolic measurements rather than co-localization alone.

Surface chemistry further determines interactions with microglia. PEGylation may reduce some nonspecific interactions but can also limit cellular entry; repeated systemic exposure raises additional concerns relating to anti-PEG antibodies and complement activation. Cell-membrane coatings introduce source-dependent proteins, lipids, orientation, and batch heterogeneity and should not be presumed immunologically inert [86], [96], [97]. In iPSC-derived microglia, candidate formulations should be compared at matched catalytic exposure for uptake, cytokine release, phagocytosis, lysosomal function, persistence, and effects on neural cells [135]. Complement- and antibody-dependent effects additionally require complement-active serum or plasma assays, with inactivated controls, because microglial monocultures do not model systemic immunity [136]. For constructs intended to act through microglia, indiscriminately minimizing uptake may conflict with the therapeutic mechanism. Fig. 4 separates barrier transport, tissue distribution, intracellular fate, and model-specific validation.

**Fig. 4:**
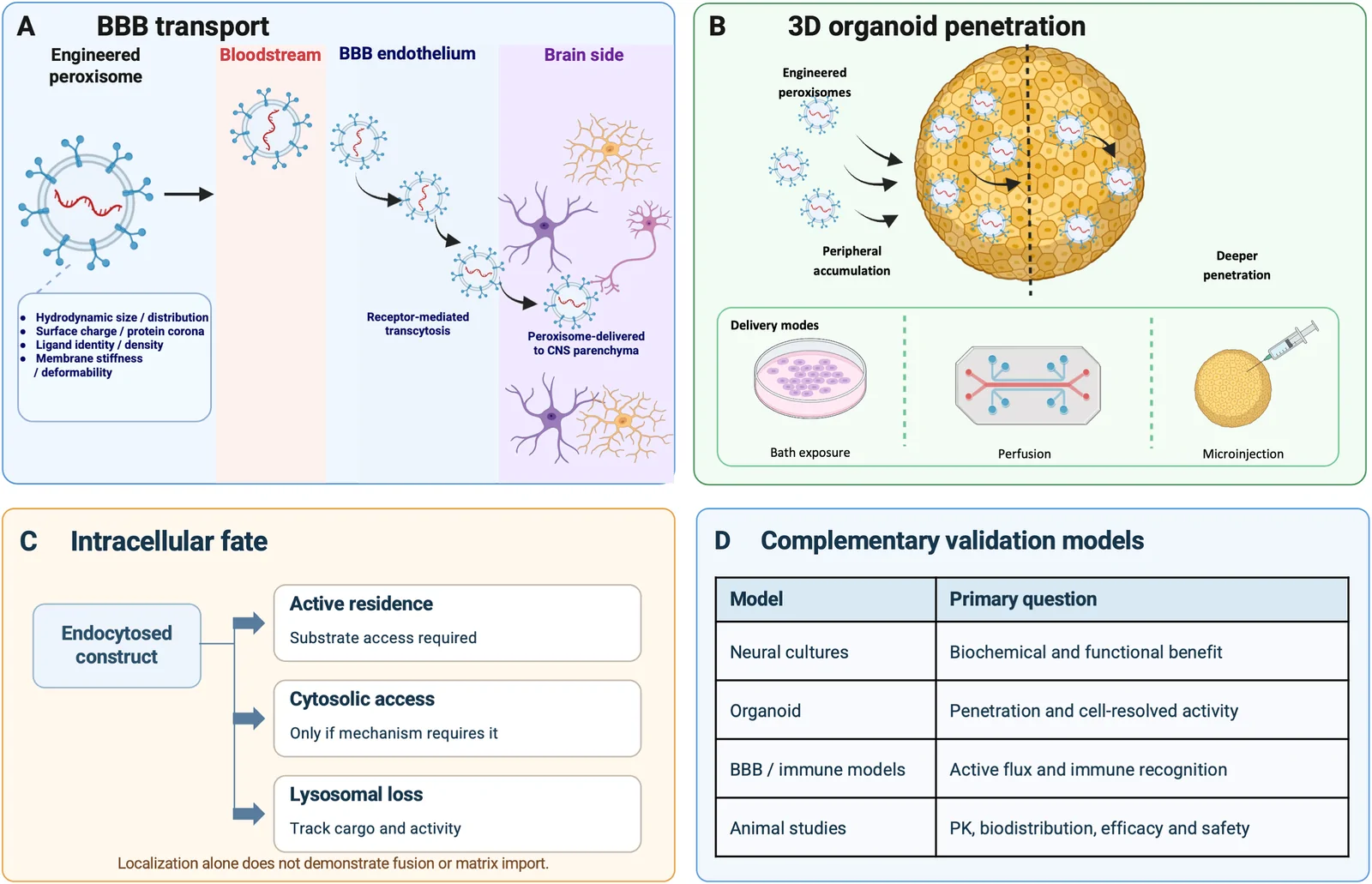
**Delivery barriers and mechanism-dependent intracellular fate.** (A) Endothelial binding and uptake must be distinguished from productive BBB transcytosis and delivery of intact active material to brain parenchyma. Size, charge, ligand density, and deformability are variables to optimize, not fixed success criteria. Red schematic particles represent enzyme-containing constructs, not nucleic-acid cargo. (B) 3D organoid exposure may produce surface accumulation and radial concentration gradients. Bath exposure, perfusion, and microinjection test different delivery constraints. (C) Endocytosed material may remain active within endosomal compartments, escape to the cytosol, or undergo lysosomal loss. The required destination depends on substrate access and mechanism; proximity to a peroxisome does not demonstrate fusion or matrix import. (D) Neural, barrier, immune, and *in vivo* models address complementary questions. Uptake, functional efficacy, penetration, immune responses, pharmacokinetics, biodistribution, and safety should be measured in the models appropriate to each endpoint. Figure was created with BioRender.com.(For interpretation of the references to colour in this figure legend, the reader is referred to the Web version of this article.)

Organoid-on-chip and multicellular neural platforms can integrate controlled delivery with neuron–glia interactions and network-level functional readouts [43], [44], [137]. Their principal value is to distinguish delivery failure from loss of cargo, inappropriate catalytic function, intracellular mislocalization, or absence of the proposed disease mechanism after delivery. Stage-resolved assays, combining carrier–cargo tracking, organelle markers, pulse-chase live imaging, depth-resolved distribution, and functional readouts, are therefore needed before a peroxisome-engineering platform is advanced or rejected [138], [139].

## 6 Clinical and translational context: peroxisomal therapies and nuclear receptor agonists

Primary peroxisomal disorders provide genetically defined contexts in which a missing biochemical function can be identified, although disease severity and developmental timing create substantial therapeutic constraints [68], [69], [75], [83], [140], [141]. Early DHA ethyl ester studies reported biochemical changes in patients with peroxisomal biogenesis disorders [142], [143]. These observations should be distinguished from controlled evidence of neurological benefit and do not validate organelle replacement. A catalase-based construct, for example, would not correct a biogenesis defect or a missing lipid pathway simply because it reduces ROS.

Plasmalogen augmentation illustrates a mechanistically distinct approach. PPI-1011 has been studied in MPTP models, including striatal dopamine outcomes in mice and dyskinesia-related outcomes in monkeys [144], [145]. A first-in-human study in healthy adults assessed safety, tolerability, and pharmacokinetics [146]. Such early clinical evidence does not establish efficacy in AD, PD, or primary peroxisomal disease. Lipid supplementation can provide a comparator when a defined ether-lipid deficit is present, but its activity should not be equated with restoration of all peroxisomal functions [147].

PPAR agonists regulate transcriptional programmes involving lipid metabolism, inflammation, and mitochondrial function, rather than acting as peroxisome substitutes [148], [149]. Preclinical outcomes can also depend on model-specific mechanisms. For example, pioglitazone protection in an MPTP model was linked to monoamine oxidase B inhibition [150], which complicates attribution to peroxisomal restoration. The phase 2 pioglitazone study in early PD did not support advancement of the tested treatment for slowing progression [151]. This outcome constrains that drug and regimen; it does not resolve the potential of all PPAR-directed strategies. Leriglitazone (MIN-102), a brain-penetrant PPARγ agonist, similarly missed its primary Six-Minute Walk Test endpoint in the ADVANCE trial in adrenomyeloneuropathy while showing reduced cerebral lesion progression and no cases of clinically progressive cerebral ALD in the treated arm. These are findings that were secondary or post-hoc and therefore hypothesis-generating rather than confirmatory [152]. Human cellular models can help compare mechanisms, but favourable in vitro results remain insufficient to predict clinical efficacy [153], [154].

Patient-derived models offer an opportunity to compare these approaches under the same biological conditions. Zellweger spectrum iPSC models demonstrate impaired peroxisome assembly and cell-type-specific lipid abnormalities [45], whereas myelinating organoid methods developed for other disorders provide a model-design precedent rather than peroxisomal efficacy evidence [50]. Comparisons should include an appropriate PPAR agonist, a lipid precursor when relevant, and a conventional antioxidant or enzyme formulation, with matched active exposure and independent functional endpoints. Whether a more complex peroxisome-inspired construct offers additional benefit is an empirical question.

## 7 iPSC-based platforms for testing peroxisome-inspired materials

### 7.1 Model selection, strengths, and limitations

Patient-derived iPSC systems differ in the questions they can answer. Undifferentiated iPSCs preserve genotype and provide a renewable source, but do not reproduce neural function. Neurons enable assessment of excitability, synaptic activity, and survival; astrocytes support testing of metabolic coupling and glutamate handling; microglia inform material uptake and innate immune responses; and oligodendrocytes are important for lipid and myelin phenotypes. Cocultures introduce neuron–glia interactions, whereas organoids add spatial organization, maturation-dependent vulnerability, and delivery constraints. However, maturation state, cellular composition, culture medium, donor-to-donor variation, and batch effects remain important limitations of these systems [21], [39], [46], [47].

Importantly, iPSC-derived neural models represent a currently unmet translational benchmarking step for engineered and artificial peroxisomes [46], [47]. However, to our knowledge, the constructs discussed in Section 4 have not yet been systematically evaluated in patient-derived iPSC neurons, astrocytes, microglia, or brain organoids from individuals with AD, PD, ALS, or KS. This gap is consequential because material properties that appear acceptable in immortalized cell lines, including vesicle or polymer-shell composition, cationic surface chemistry, and residual synthesis by-products, may cause cytotoxic, developmental, or sublethal functional effects in long-term post-mitotic neural cultures [155]. In addition, delivery into densely packed organoid tissue is less predictable than delivery into monolayer cultures, because particle penetration and cellular uptake are strongly influenced by particle size, surface chemistry, charge, mechanical properties, extracellular-matrix architecture, and spatially heterogeneous diffusion [124]. These parameters therefore require direct validation in each relevant model system.

Table 4 matches each model to its most informative application and interpretation limits. Biological replication should include independent donors when the claim concerns a patient population, together with independent differentiation batches to assess reproducibility. Multiple wells or organoids generated from a single differentiation should not be treated as independent donor samples. Isogenic correction or introduction of a disease-associated variant can strengthen causal interpretation, but should be complemented by additional genetic backgrounds where feasible. Analyses should account for donor and batch structure, report exclusions transparently, and avoid treating technical replicates as independent biological samples.

**Table 4:** Functional comparison of peroxisome-related therapeutic platform concepts.

Platform	Composition and cargo	ROS handling and lipid function	Targeting and uptake	Transport or localization	Potential advantage	Principal limitation	Developmental status
**Natural biological peroxisome**	Endogenous organelle with native membrane, transporters and metabolic enzymes [63], [65].	Both oxidant production and detoxification; pathway-specific lipid metabolism.	Endogenous organelle maintenance differs from uptake of an isolated organelle.	Native protein import and metabolite exchange; integrity may change after isolation.	Biological architecture and a broad endogenous enzyme repertoire.	Isolation purity, viability of cargo, functional stability, delivery and batch variability.	Native biology is established; CNS therapeutic transplantation is not established by the cited evidence.
**Engineered biological peroxisome**	Biological organelle modified genetically or chemically; cargo is design-dependent [84], [91].	Intended modification of selected native functions; retained activity must be measured.	*In situ* modification and exogenous delivery are different interventions.	Biological membrane and localization machinery, subject to engineering-related changes.	Can retain organelle architecture while modifying a defined function.	Perturbation tools are not replacements; complexity and delivery remain major barriers.	Experimental tools and platform concepts; no integrated patient-derived CNS efficacy benchmark here.
**Membrane-bounded artificial peroxisome**	Lipid, polymer or hybrid compartment with specified enzymes or catalysts [19], [85], [92], [93], [94].	Selected catalytic reactions. Antioxidant loading does not establish VLCFA or plasmalogen pathway restoration.	Endocytosis with optional ligands; specificity requires matched controls.	Permeability must permit substrate access and retain active cargo; intracellular destination is mechanism-dependent.	Control of composition, transport, active loading and surface chemistry.	Transport limitation, leakage, endolysosomal fate, immune effects and incomplete biomimicry.	Preclinical artificial-organelle evidence; integrated AD/KS iPSC neural validation remains unestablished in the examples reviewed.
**Non-vesicular peroxisome-mimetic assembly**	Catalytic material in a programmed scaffold, exemplified by DNA–ceria [87].	Enzyme-like redox catalysis; no complete native lipid network.	Uptake and intracellular assembly can control retention.	No native peroxisomal membrane or matrix-import machinery.	Programmable assembly and persistent selected catalytic activity.	Catalyst persistence, assembly heterogeneity and limited correspondence to a natural organelle.	Cellular proof of concept; CNS therapeutic efficacy requires separate evidence.
**Conventional antioxidant nanocarrier**	Lipidic, polymeric, inorganic or hybrid carrier containing a drug, enzyme or nanozyme [156], [157], [158].	Delivery or catalysis of selected antioxidants; activity may overlap with artificial-peroxisome constructs.	Established carrier-design approaches; CNS selectivity is formulation-specific.	Release, cellular uptake or local catalysis, depending on mechanism.	Potentially simpler formulation and a useful matched comparator.	Uncontrolled exposure, nonspecific effects or limited retention may still occur.	Maturity varies widely by product; nanocarrier precedent does not establish efficacy of a CNS peroxisome-inspired system.

Note: Biomimetic describes a reproduced biological feature and may apply to several rows. Nanoorganelle describes a nanoscale functional construct, not proof of peroxisomal identity. No common quantitative ROS-scavenging rank is assigned: enzyme activity, dose, substrate, exposure, and assay conditions differ.

Abbreviations: CNS, central nervous system; VLCFA, very-long-chain fatty acid; AD Alzheimer's disease; KS, Kleefstra syndrome; iPSC, induced pluripotent stem cell.

A staged evaluation should first establish a disease-associated phenotype under baseline conditions, then determine whether the proposed material engages its intended biochemical target [46], [47], [159]. Neurons, glia, cocultures, and three-dimensional models should be selected according to the proposed mechanism and delivery route rather than arranged as a mandatory progression in every study. BBB models and systemic immune assays may be performed alongside neural efficacy studies when they address distinct translational risks, such as endothelial transport, peripheral clearance, or immunogenicity.

Chemical challenges serve different purposes. H_2_O_2_ exposure can test peroxide-buffering capacity, whereas rotenone, MPP^+^, or paraquat can probe mitochondrial or redox-cycling vulnerability; however, none of these agents constitutes a peroxisome-specific stressor. Likewise, disease-associated protein assemblies, excitotoxicity, and inflammatory stimuli require careful control of preparation, dose, duration, and cell composition. A challenge concentration should not be described as disease-relevant solely because it increases ROS levels in culture. Table 5 distinguishes published exposure examples from proposed calibration conditions and identifies the controls needed for their interpretation. Baseline disease phenotypes and chronic low-intensity challenges should accompany acute capacity testing whenever feasible [42], [159], [160].

**Table 5:** Oxidative-stress paradigms and controls for neural biomaterial evaluation.

Paradigm and purpose	Exposure or calibration	Disease context	Evidence status	Essential controls	Useful endpoints	Limit on interpretation
**Baseline disease phenotype**	No exogenous stress. Define differentiation stage, medium composition and sampling times before treatment.	-	Proposed first step in all disease-specific evaluations.	Matched control donors, isogenic comparison when available, vehicle and empty material.	Peroxisomal import and lipid function, redox state, survival and neural function.	A baseline disease difference must be reproducible before assigning disease-specific rescue.
**Acute H_2_O_2_ challenge**	Example pilot screen: 1–100 μM for 0.5–2 h, followed by washout and 24–48 h observation. Recalibrate for each model.	AD, PD, ALS (acute injury modeling)	Proposed calibration range, not a validated universal protocol or an estimate of brain H_2_O_2_ exposure.	Stress alone, extracellular free catalase, matched active cargo, empty material, catalytically inactive comparator if physically matched.	Peroxide handling, oxidative damage, recovery and viability; carrier/cargo integrity.	Can favour extracellular scavenging. Protection during co-exposure does not prove intracellular delivery.
**Paraquat toxicant sensitivity**	72 h exposure; differences at 10 and 30 μM; reported EC50 4.8 μM (KS) and 23.7 μM (control) [5].	KS coritcal neurosphere	Published model-specific anchor. These are sensitivity results, not recommended chronic treatment concentrations.	Matched neuronal maturity and spheroid size, control genotype, vehicle and cell-free assay controls.	Dose–response viability and oxidative injury with orthogonal functional measures.	Redox cycling is not peroxisome-specific. A single patient/control comparison does not establish population-level KS biology.
**Rotenone challenge**	72 h; differences at 0.01 and 0.1 μM; reported EC50 0.11 μM (KS) and 0.23 μM (control) [5].	KS neurosphere	Published compound- and maturation-dependent sensitivity example.	Solvent control, matched maturation, stress alone, antioxidant and mitochondrial mechanism controls.	Respiratory function, mitochondrial potential, oxidative damage and survival.	Complex I inhibition does not establish a primary peroxisomal defect.
**Gradual antioxidant withdrawal**	Published example: B27 minus antioxidants in DMEM/F12; longitudinal neurite imaging for 13 days, with 14-day screening [42].	AD, PD, ALS (aging-associated modeling)	Healthy-donor iPSC-derived neuron model, not a patient-derived ALS efficacy validation.	Matched antioxidant-containing medium, matched cell density and medium exchange, positive rescue comparator.	Longitudinal neurite response, lipid peroxidation, survival and mechanism-specific rescue.	Medium and cell density strongly affect injury; report and verify the actual plating density.
**Disease-associated protein or inflammatory challenge**	Use a model-specific dose–time matrix. Specify protein species and assembly state, or cytokine/LPS composition. No universal concentration is assigned.	All contexts (multifactorial modelling)	Proposed model-dependent validation; disease relevance must be demonstrated.	Preparation/vehicle controls, appropriate inactive or nonpathological comparator, composition-matched coculture and targeting controls.	Cell-specific injury, inflammatory response, neural activity and peroxisomal function.	A generic inflammatory or aggregation assay cannot establish a disease-specific peroxisomal mechanism.
**Peroxisome-specific perturbation and rescue**	Choose a defined biogenesis, transport or enzyme defect and a calibrated genetic/biochemical perturbation. Avoid a universal knockdown level.	KS (intrinsic vulnerability)	Proposed causal test, informed by genetically defined models [45].	Isogenic correction or rescue, non-targeting control, pathway-appropriate lipid or enzyme comparator.	Protein import, relevant lipid flux, compartment-specific redox response and neural function.	Marker expression alone is insufficient; rescue should link the biochemical defect to function.

Note: All exposures are in vitro. Published examples and proposed pilot ranges are labelled separately. Concentration, duration, medium, maturity, model geometry and recovery determine the biological exposure. For every material: report both material amount and active catalytic units, use empty and ligand-free controls where relevant, and test optical/chemical assay interference. A lower ROS-probe signal alone is not efficacy. For preloading experiments, wash away extracellular material before challenge when testing intracellular protection, and compare with simultaneous addition. Confirm retained intracellular activity.

Abbreviations: AD, Alzheimer's disease; PD, Parkinson's disease; ALS, Amyotrophic lateral sclerosis; KS, Kleefstra syndrome; iPSC, induced pluripotent stem cell; LPS, lipopolysaccharide.

Future peroxisome-inspired materials may also extend beyond enzyme encapsulation and surface-ligand functionalization. For example, protein–protein interaction-targeting design principles, including self-assembling organelle-selective peptide amphiphiles, could potentially be adapted to modulate peroxisomal docking, membrane contacts, matrix-protein import, or other interactions relevant to peroxisome biogenesis and peroxisome–mitochondria cross-talk [90]. Such concepts remain speculative in the peroxisomal context and require careful validation of target engagement, selectivity, and functional consequences in human neural cells [90], [160], [161].

### 7.2 Recommended experimental logic for redox challenge paradigms

The proposed experimental sequence begins with baseline profiling of redox status, lipid oxidation, mitochondrial function, and peroxisomal abundance and activity, followed by dose–time calibration and mechanistic rescue experiments. Acute stress paradigms may include H_2_O_2_ at approximately 25–200 μM for 1–24 h, glutamate at approximately 100 μM–1 mM for 30 min–24 h, or mitochondrial complex I inhibitors such as rotenone at 10 nM–1 μM or MPP^+^ at 1–50 μM for 24–72 h. These conditions should be treated as starting ranges for cell type- and line-specific calibration, rather than universal protocols. The selected exposure should produce reproducible sublethal or moderately injurious phenotypes within a predefined dynamic range, allowing both worsening and rescue to be detected [42], [159], [162].

For late-onset NDD models, chronic low-grade paradigms are generally more appropriate than acute high-dose insults for testing whether peroxisome-inspired materials slow or reverse progressive degeneration. Examples include repeated low-dose H_2_O_2_ exposure at approximately 5–25 μM over 5–14 days, sustained low concentrations of mitochondrial inhibitors, or gradual depletion of endogenous antioxidant capacity, for example using buthionine sulfoximine at approximately 50–100 μM for 3–7 days. Because H_2_O_2_ added as a bolus is largely consumed within minutes by endogenous catalase and glutathione peroxidase, repeated low-dose dosing models recurrent oxidative episodes rather than sustained exposure; a maintained elevation requires a generating system (for example glucose oxidase) or a continuous stressor such as low-dose mitochondrial inhibition or glutathione depletion. This distinction matters for calibration, since the two regimens test tolerance of transient peroxide spikes and performance under continuous load, respectively. Where possible, such challenges should be applied to relatively mature neuronal cultures, ideally after prolonged differentiation, because maturation-dependent metabolic demands and vulnerability are likely to influence treatment responses. In AD and PD models, these paradigms may be used to assess whether candidate materials preserve neuronal survival, organelle function, synaptic integrity, and network activity under sustained oxidative burden [42], [48], [49]. In ALS, where direct disease-specific evidence for peroxisomal dysfunction remains comparatively limited, iPSC-derived motor-neuron and glial cultures should initially be regarded as hypothesis-testing platforms to determine whether peroxisomal abnormalities contribute causally to oxidative injury, rather than as systems in which therapeutic efficacy can yet be predicted confidently [42].

For KS, the key mechanistic question is whether a reproducible peroxisomal abnormality can first be demonstrated and then selectively corrected. Because direct evidence of peroxisomal dysfunction in KS remains limited, endogenous baseline monitoring across defined stages of neural or cortical differentiation, benchmarked against isogenic and unaffected donor lines, may be more informative than simply superimposing an exogenous oxidative challenge. Relevant assays should assess peroxisomal metabolism and integrity directly, for example through VLCFA processing, ether-lipid composition, matrix-protein import, peroxisomal enzyme activity, organelle abundance, or compartment-specific redox measurements. LDHA transcript changes should not be conflated with extracellular LDH release, which is primarily a membrane-damage readout. Multiple donor backgrounds and isogenic comparisons are needed before a material response can be interpreted as KS-specific [5], [7], [27].

Essential comparisons include untreated and vehicle controls, stress alone, empty material, free enzyme or catalyst at matched activity, and an appropriate conventional antioxidant rescue control. N-acetylcysteine or Trolox, for example, may serve as positive redox-rescue controls when dose-optimized in the relevant model. Isogenic CRISPR-corrected lines, where available, provide particularly valuable genetic controls. A catalytically inactive construct can test catalytic dependence, provided that inactivation does not substantially alter the construct's size, surface chemistry, stability, or other material properties. Ligand-free and receptor-blocked conditions can help assess targeting specificity. Material-only, probe-only, and cell-free interference controls are also essential because particulate or nanostructured materials may adsorb fluorescent dyes, scatter light, quench signals, or alter assay chemistry. Consequently, a reduction in a fluorescent ROS signal alone should not be interpreted as evidence of neuroprotection or peroxisomal rescue [42], [159], [162].

Candidate constructs should be dosed using both material amount and active catalytic units, with exposure duration, colloidal stability, enzyme activity, and medium stability reported explicitly. Carrier and cargo should be tracked separately where possible. Any reduction in oxidative damage should be interpreted in relation to the amount of intact, active material reaching the intended cellular compartment, rather than solely to the nominal amount added to the culture well. In organoids, this requirement is especially important because limited penetration or preferential uptake by peripheral cells may create misleading bulk readouts [156], [157].

Mechanistic readouts should match the claimed function. Peroxide handling requires species- and compartment-aware assays, while peroxisomal rescue requires direct measures of peroxisomal metabolism rather than expression of a peroxisomal marker alone. Mitochondrial bioenergetic analyses and live-cell imaging can assess downstream inter-organelle consequences, including peroxisome–mitochondria contacts, organelle distribution, respiratory performance, and β-oxidation-related metabolic function [77], [163], [164]. Orthogonal measures of oxidative damage, lipid peroxidation, ferroptosis-related pathways, and cellular function should accompany ROS reporters. In particular, ferroptotic cell death should be assigned using functional and pathway-specific evidence rather than lipid-peroxidation measurements alone. Neurite structure, synaptic density, calcium dynamics, multielectrode-array activity, and electrophysiological recordings can then establish whether biochemical rescue is accompanied by meaningful neural benefit [42], [48], [49].

Single-agent chemical stressors, even when rigorously calibrated, only partially reproduce the chronic and multifactorial oxidative stress of human neurological disease, which reflects interacting proteotoxic, inflammatory, metabolic, and organelle-level disturbances accumulated over years or decades. More disease-relevant validation should therefore incorporate combinatorial and extended-duration paradigms where feasible. Examples include low-dose H_2_O_2_ combined with pro-inflammatory cytokines such as TNF-α or IL-1β, mitochondrial stress combined with disease-associated protein assemblies, or neuron–glia cocultures in which microglial activation provides an endogenous inflammatory component. These more complex models are resource-intensive but may better distinguish genuinely disease-modifying constructs from materials that merely suppress an acute chemical ROS signal [42], [162].

These platforms can support candidate prioritization, dose–response analysis, combination-treatment studies, and identification of biomarkers that may guide later translational development [159], [165], [166], [167], [168]. Their predictive value, however, depends on the biological relevance of the selected model, rigorous control of exposure and assay artefacts, and reproducibility across donors and differentiations. A peroxisome-inspired construct should advance only when it produces an interpretable benefit that cannot reasonably be explained by assay interference, nonspecific stress suppression, unequal cellular exposure, or unrecognized material toxicity [159], [166].

## 8 Surfaceome-guided targeting and biomarker discovery

The cell-surface proteome can connect a disease-associated cellular state with candidate delivery targets. However, a useful disease biomarker, an accessible surface antigen, an internalizing receptor, and a BBB transport receptor are different functional categories. A marker identified in culture does not automatically satisfy all of them [100], [158], [169].

Surfaceome profiling of patient-derived neural populations can identify accessible membrane proteins for subsequent validation [170], [171], [172], [173]. Glycoprotein enrichment and mass spectrometry provide information complementary to RNA abundance, but labelling efficiency, viability, and intracellular contamination require control. In mixed cultures, apparent differences may reflect cell composition rather than altered expression within a cell type. Neural glycoproteomic studies support the feasibility of this approach [174], [175], but do not establish a validated KS-specific delivery receptor.

Candidate prioritization can combine extracellular accessibility, protein abundance, differential expression, and functional internalization. Computational surfaceome resources and oncology examples demonstrate discovery workflows [176], [177], while neurological nanomedicine provides delivery precedents [178], [179]. These are transferable methods, not proof that the same targets will direct peroxisome-inspired materials to diseased CNS cells. Epigenetic and transcriptional changes in Kabuki syndrome likewise motivate comparison with KS but do not demonstrate a shared surface target [180].

### 8.1 Translating surfaceome discovery into selective CNS targeting

Illustrative candidates include NCAM1 or L1CAM for neuronal populations, SLC1A3/GLAST for astrocyte-enriched populations, TREM2 or CX3CR1 for microglia, and MOG for mature oligodendrocytes and myelin. These are starting points for testing, not validated universal delivery receptors. NCAM1 and L1CAM are not restricted to the CNS, CX3CR1 also occurs in peripheral immune populations, and TREM2-dependent uptake can alter signalling. SLC1A3 accessibility does not establish productive internalization, while MOG binding can introduce immune and myelin safety concerns. Disease state, maturation, ligand-induced signalling, and peripheral expression must therefore guide selection [120], [181], [182], [183].

A targeting workflow should validate surface accessibility and enrichment with an independent assay, determine internalization and intracellular routing, and compare uptake across relevant neural and peripheral populations. Receptor competition or genetic perturbation can test mechanism. Whole-blood and serum exposure should be included when relevant because the protein corona can mask ligands. BBB transport and post-BBB cell targeting may require distinct design features; a ligand that binds neurons is not necessarily a BBB shuttle. Off-target uptake in endothelium, liver-associated cells, and circulating phagocytes needs direct assessment rather than inference from an iPSC-only comparison [176], [184], [185].

Disease-associated glial states may provide candidate markers in neurodegeneration [39], [186], [187], whereas developmental models require attention to changes in maturation and cell proportions. A target should be selected only when the relevant surface state is reproducible and the consequences of receptor engagement are acceptable. Differential abundance alone is insufficient.

Antibodies, peptides, or receptor ligands can be considered after functional validation. Ligand density and orientation should be measured, and their effects compared with otherwise matched ligand-free constructs. Selective binding, uptake, and biochemical activity are separate outcomes; none should be inferred solely from the presence of a targeting moiety [85], [188].

Longitudinal surface profiling may help identify adaptive responses to treatment and refine candidate ligands [189]. It should be interpreted together with cell viability, cell composition, trafficking, and functional endpoints. This creates an experimental feedback process rather than a guarantee of patient stratification or clinical benefit. Evidence from tumour surfaceomes or non-neural iPSC systems can inform methods, but requires independent validation in the intended CNS model [95], [190], [191], [192], [193].

## 9 Safety, manufacturing, and realistic translational development

Safety assessment should extend beyond short-term viability. Relevant risks include complement activation, innate or adaptive immune responses, enzyme leakage, altered lysosomal function, off-target tissue accumulation, and persistent catalytic activity [97]. Material composition introduces additional questions: polymer degradation can alter the local chemical environment, inorganic catalysts may persist, and membrane coatings can carry biologically active proteins or lipids. Porins and other protein components require assessment of immunogenicity and contamination. Repeated exposure is especially relevant to chronic CNS indications. Neural and systemic assays should evaluate both the active construct and its degradation products at exposure levels supported by pharmacokinetic measurements.

Excessive oxidant removal can interfere with normal redox-dependent signalling, metabolic adaptation, and immune function. The target is therefore an exposure range that improves a disease-associated defect while preserving baseline cellular function [17], [82], [115]. Catalytic loading and trigger responsiveness can be adjusted, but their safety advantage requires measurement. The recent reviews on PANoptosis and on autophagy, ferroptosis, and neuroinflammation [194], [195] provide context for interconnected injury pathways, not evidence that an artificial peroxisome modulates those pathways therapeutically. Similarly, dose-dependent and endpoint-dependent fingolimod effects in an STZ rat model [162] illustrate why efficacy and tolerability must be assessed together, without establishing an iPSC or peroxisome-specific mechanism.

### 9.1 Developmental sequence from material optimization to clinical testing

Development should proceed through explicit decision points (Fig. 1). Materials characterization must establish composition, size distribution, morphology, surface properties, permeability, retained active cargo, and stability. Cellular work should then demonstrate mechanistic activity, distinguish assay interference, and define a usable exposure range. Patient-derived neural validation should establish reproducibility and functional benefit in an appropriate model. Delivery studies should demonstrate intact material and catalytic activity beyond the relevant barrier and in the intended target population. Animal studies must relate biodistribution, pharmacokinetics, clearance, dose-response, and efficacy, with repeat-dose toxicology appropriate to the intended route and duration. Manufacturing development and regulatory consultation should begin early and continue alongside these stages. First-in-human consideration depends on a coherent benefit-risk package; success in an organoid alone is insufficient.

Manufacturing controls should link critical material attributes to biological performance. Candidate release specifications include identity and composition, particle and aggregate distributions, active enzyme content, residual free enzyme, ligand density, membrane integrity, and a mechanism-linked potency assay. Sterility or microbiological controls, endotoxin, residual reagents, storage conditions, and stability after administration also require attention. For biological membranes, source, consistency, and preservation of relevant surface proteins need control. Acceptance limits should be justified for the final product rather than borrowed from an unrelated nanoparticle platform. Regulatory classification depends on composition, mechanism, route, and jurisdiction and should not be assumed from the term nanoorganelle.

### 9.2 Relationship to plasmalogen replacement and PPAR-directed therapies

PPAR agonists, lipid replacement, and peroxisome-inspired catalytic materials address different mechanisms. PPAR agonists modify transcriptional programmes, plasmalogen precursors supply components of a lipid pathway, and artificial catalytic systems add a selected reaction capacity. They may become alternatives or complements in a defined disease context, but complementarity and synergy are not established by combining them. Comparative studies should determine whether an intact endogenous pathway is required, whether a specific product is deficient, and whether compartmentalized catalysis adds benefit. Increased formulation complexity is justified only if it improves a clinically relevant property.

## 10 Conclusions and future perspectives

Peroxisomal functions are essential to neural biology, but their therapeutic relevance differs across disorders. Genetic and biochemical evidence establishes causality in primary peroxisomal diseases. In AD, PD, and ALS, the significance of reported abnormalities ranges from potentially contributory to unresolved. KS offers a model for testing a peroxisomal hypothesis rather than evidence of a peroxisome-driven disorder. Recent work has broadened the biological framework of Kleefstra syndrome beyond a neuron-centred model. In human iPSC-derived systems, EHMT1^+/−^ astrocytes contributed directly to neuronal network dysfunction via astrocyte-dependent mechanisms linked to increased S100B signalling [196], while Drosophila lacking G9a, the functional orthologue of EHMT1, showed impaired oxidative stress tolerance with excessive glycogen depletion and defective lipid energy mobilization [197]. Neither study demonstrates a primary peroxisomal defect, but both implicate processes that overlap with established peroxisomal functions, including redox regulation, lipid metabolism, and metabolic stress responses. This strengthens the rationale for investigating peroxisome-related pathways in patient-derived KS models, while a causal link between EHMT1 haploinsufficiency and peroxisomal dysfunction remains to be established experimentally.

Artificial-organelle studies demonstrate selected catalytic, transport, and targeting principles. They do not yet validate the integrated patient-derived CNS strategy discussed here. Patient-derived neural cultures can test mechanistic activity, cell interactions, and functional outcomes, while their incomplete maturation, donor variability, and transport constraints must remain part of interpretation.

The next decisive step is to demonstrate that a defined material corrects a measured biochemical defect and improves neural function at a verified intracellular exposure. This requires matched comparators, quantitative transport and activity measurements, and evidence that normal redox signalling is preserved. BBB delivery, tissue penetration, biodistribution, repeated-dose safety, and reproducible manufacturing remain independent requirements.

Comparative evaluation against simpler antioxidants, plasmalogen-directed approaches, and PPAR modulation will determine where peroxisome engineering offers a meaningful advantage. Its present value is a testable biomaterials framework. Clinical relevance will depend on reproducible disease-specific benefit and an acceptable benefit-risk profile, rather than on organelle mimicry or ROS reduction alone.

## CRediT authorship contribution statement

**Csaba Matta:** Writing – review & editing, Writing – original draft, Supervision, Project administration, Investigation, Funding acquisition, Conceptualization. **Yoon-Hee Cheon:** Writing – review & editing, Writing – original draft, Investigation. **Judit Vágó:** Writing – review & editing, Writing – original draft, Investigation. **Zoltán Mészár:** Writing – review & editing, Writing – original draft, Investigation. **Ee-Hyun Kim:** Writing – review & editing, Writing – original draft, Visualization, Investigation. **Songling Jiang:** Writing – review & editing, Writing – original draft, Investigation. **Andrea Balogh:** Writing – review & editing, Writing – original draft, Investigation. **Melinda Zana:** Writing – review & editing, Writing – original draft, Investigation. **András Dinnyés:** Writing – review & editing, Writing – original draft, Supervision, Project administration, Investigation, Funding acquisition, Conceptualization. **Eun-Jung Jin:** Writing – review & editing, Writing – original draft, Supervision, Project administration, Funding acquisition, Conceptualization.

## Declaration of generative AI and AI-assisted technologies in the manuscript preparation process

During preparation of this work, the authors used Perplexity for language editing and ChatGPT for editorial revision and consistency checking. The authors are responsible for reviewing the revised text, figures, tables, and citations and for the scientific content of the published article.

## Declaration of competing interests

The authors declare that they have no known competing financial interests or personal relationships that could have appeared to influence the work reported in this paper.

## Data Availability

No data was used for the research described in the article.

## Abbreviations

AD: Alzheimer's disease
ALS: amyotrophic lateral sclerosis
BBB: blood-brain barrier
CAT: catalase
CNS: central nervous system
CPP: cell-penetrating peptide
DHA-EE: docosahexaenoic acid ethyl ester
ECM: extracellular matrix
iPSC: induced pluripotent stem cell
KS: Kleefstra syndrome
LDHA: lactate dehydrogenase A
LPO: lactoperoxidase
MECP2: methyl-CpG-binding protein 2 gene
NDD: neurodegenerative disorder
PCL: poly(ε-caprolactone)
PD: Parkinson's disease
PDMS: poly(dimethylsiloxane)
PDPA: poly(2-(diisopropylamino)ethyl methacrylate)
PEG: poly(ethylene glycol)
PLA: poly(lactic acid)
PLAAT: phospholipase A and acyltransferase
PMOXA: poly(2-methyl-2-oxazoline)
PMPC: poly(2-methacryloyloxyethyl phosphorylcholine)
PPAR: peroxisome proliferator-activated receptor
ROS: reactive oxygen species
RTT: Rett syndrome
SOD: superoxide dismutase
VLCFA: very-long-chain fatty acid
